# Situations of work-related diseases and injuries among agriculturists in the upper northeast regions of Thailand

**DOI:** 10.12688/f1000research.73221.1

**Published:** 2022-02-04

**Authors:** Sunisa Chaiklieng, Chuthamas Chagkornburee, Pornnapa Suggaravetsiri

**Affiliations:** 1 Department of Environmental Health, Occupational Health and Safety, Faculty of Public Health, Khon Kaen University,, Khon Kaen, 40002, Thailand; 2 Master of Science Program in Occupational Health and Safety, Faculty of Public Health, Faculty of Public Health, Khon Kaen University, Muang Khon Kaen, Khon Kaen, 40002, Thailand; 3 Department of Epidemiology and Biostatistics, Faculty of Public Health, Khon Kaen University, Khon Kaen, 40002, Thailand

**Keywords:** Big data, occupational disease, agriculturists, surveillance, ICD-10

## Abstract

**Background: **Agriculturists exposed to health hazards are affected by increased occupational disease. This retrospective study aimed to investigate situations of work-related diseases and injuries among agriculturists in upper northeast Thailand.

**Methods: **The secondary data of international classification of diseases 10 ^th^ revision (ICD-10) case reports of occupational disease among farmers for 2014-2016, from the database of the Health Data Center (HDC), were used. We collected the number of registered farmers from the agricultural office and secondary data of ICD-10 from the hospital information system (HIS) of healthcare services in Udon Thani and Roi-Et provinces. The annual morbidity rate of occupational diseases was analyzed and presented at a rate per 100,000 farmers of the studied area.

**Results: **Among farmers who visited healthcare service providers, lung disease, which was not reported as occupational disease of the HDC database, was the highest ranking of all diseases, followed by work-related musculoskeletal disorders (WMSDs), noise- and heat-related diseases, and pesticide toxicity, respectively, while the injury rate was as high as that of WMSDs. The morbidity rate of Udon Thani was higher than that of Roi-Et province, whose rate was closer to the national rate. The number of farmers from the HDC database was not reflective of numbers of registered farmers, even though the case reports of the HDC was lower than that of the HIS with occupational ICD-10 codes, which might explain the underestimation of the morbidity rate of occupational disease.

**Conclusions: **The morbidity rate of lung disease, noise- and heat-related diseases, and pesticide toxicity increased every year (2014-2016) and reflects the health problems among agriculturists in Thailand. The underestimation in the reported disease rate is explained from big data analysis by the rare recording of work-related cases among those agriculturists. Therefore, Thai agriculturists should be supported in registration with occupational diseases surveillance as holistic healthcare.

## Introduction

From the survey results in 2019, it was found that the total number of employed persons in Thailand was 37.5 million persons. The number of workforce members who had no social security or were informal workers was about 20.4 million, or 54.3%. By region, those in informal employment working in the Northeast made up the largest proportion (34.9%), followed by Central Thailand (23.4%), the North (20.9%), and the South (14.0%). ^1^ When the economic activities of those in informal employment were considered, it was found that more than half of all informally employed persons worked in the agriculture sector (about 11.5 million, or 56.4%). ^1^

Agriculturists exposed to health hazards are affected by increased occupational disease. According to a global report on occupational diseases, the highest prevalence of such disease was work-related musculoskeletal disorders (WMSDs), which ranged between 55.6-97.2%. ^2^^–^^4^ The highest reported prevalence was in Korea (97.2%), ^2^ followed by Saskatchewan province of Canada (85.6%), ^3^ and Ireland (55.6%). ^4^ The next most prevalent disease was heat-related disease, which ranged from 35.6-72.3%. ^5^^–^^8^ The highest reported prevalence was found in Eastern North Carolina, USA, ^5^ followed by Northeast Italy, ^6^ and North Carolina, USA. ^7^^,^^8^ Additionally, prevalence of hearing loss from noise was reported at levels of 16.9-36.1%, ^9^^,^^10^ which were 16.9% ^9^ in Korea and 36.1% ^10^ in the USA, while work-related injuries were reported at a prevalence of 69.0% in Nepal. ^11^ Infectious disease was reported in ranges between 0.7-13.5%, ^12^^–^^14^ where brucellosis cases in Greek farmers and livestock breeders corresponded to an annual incidence rate of 7.1 per 100,000 population, ^15^ and 8.8-17.0%, which were 8.8% in China, ^16^ 8.9% in the Western Cape of South Africa, ^17^ and 17.0% in the Sunsari District of Nepal. ^18^ Chronic lower respiratory tract disease was also reported as chronic obstructive pulmonary disease (COPD) and was found in a systematic review to be from 3.0% to 68.0%, ^19^ as in France, where the rate of COPD was 9.5%, ^20^ and South Africa, where the rate was 3.0% for asthma. ^21^

In Thailand, the health database of the Health Data Center (HDC) ( https://hdcservice.moph.go.th/hdc/main/index.php) showed that the highest prevalence of disease between 2014-2016 was for work-related injuries (about 0.6%), followed by WMSDs (about 0.3%), neurotic hearing loss from noise and heat-related disease (0.2%), and pesticide toxicity (0.04%). There was no statistical report on work-related diseases which included respiratory symptoms or lung disease, and infectious disease on the health database of the HDC. In 2019, the prevalence of chronic lower respiratory tract disease was reported of 15-59 years old population to be 1.8%. ^22^ The previous five-year retrospective study showed a higher morbidity rate of all cases in the research from the Nongbualamphu province of Thailand, which found the highest incidence was for WMSDs (21.7%), followed by pesticide toxicity (0.18-42.9%), ^23^^–^^25^ which can be compared to the three-year incidence rate of 48.48 per 100,000 farmers or 0.05%. ^26^ The study also indicated a rate of infectious disease at 0.51%, ^23^ with chronic lower respiratory tract disease (1.11%) and skin disease (1.5%) ^23^ as additional diseases which occurred. The case-control study in agriculturists of Khon Kaen found that noise-induced hearing loss was 49.7%. ^27^ Work-related injuries had a prevalence of 20.1% among the farmers of the Phayao province ^28^ and the prevalence of heat-related disease was also reported as 13.5 per 100,000 farmers, or 0.014%, in agriculturists of the Khon Kaen province. ^29^

Those work-related diseases and injuries in Thailand were identified by the International Statistical Classification of Diseases and Related Health Problems 10 ^th^ revision (ICD-10) ^30^ ( https://icd.who.int/browse10/2019/en) and the specified Y96 external code (work related condition). Nowadays, however, some cases of agriculturists are not practically identified, and this leads to inconsistency in data as above. Hence, healthcare for agricultural workers in Thailand is not covered by labor legislation and as a result they only access services from the National Health Security Office (NHSO) ( https://eng.nhso.go.th/view/1/Home/EN-US), without claiming compensation for diseases related to agricultural activities. Although occupational disease case reports are important for enabling the surveillance system to carry out national health policy among agriculturists, there is still no representative morbidity rate of the upper northeast of Thailand. Therefore, this study aimed to investigate situations of work-related diseases and injuries among agriculturists in the upper northeast of Thailand by using the case study of the Udon Thani and Roi-Et provinces.

## Methods

### Ethical approval

This study was approved by the Human Research Ethics Committee of Khon Kaen University (No. HE592154). The underlying data for this study is secondary data collected by the Health Data Centre and agricultural registries and is restricted due to ethical and data protection considerations. Under the ethical approval from the Human Research Ethics Committee of Khon Kaen University (No. HE592154), participants’ data was not allowed to be shared and the secondary data of health dataset and registered farmer dataset were utilized under restriction. The individual information or further reused inclusion data is not permitted under the ethically approved conditions for human research. This was confirmed under the data protection of human/participant privacy as no individuals gave explicit written consent that their identifiable data can be made publicly available.

### Study design

This study was a retrospective analytic study that used secondary data from three sources, which were 1) The health database of the Health Data Center (HDC) of Thailand, between 2014 and 2016, for the number of cases of occupational disease among agriculturists (by occupational code) who visited public healthcare service providers as details in HDC data collection and public health region seven (PH region 7) and public health region eight (PH region 8) in Thailand; 2) The secondary data of the list of cultivating farmers who had registered with the provincial agriculture offices of Roi-Et ( http://www.roiet.doae.go.th/) and Udon Thani provinces ( http://www.udonthani.doae.go.th/) between 2014 and 2016 as in the report Form 04-2; and 3) The secondary data of ICD-10 utilized from the hospital information system (HIS) database as detailed in the HIS data collection that used for routine care services in primary care units, secondary, and tertiary hospitals was selected from the provinces of the pilot study areas (Roi-Et and Udon Thani).

### Population and sample size

The sample size of the retrospective descriptive study was the total number of registered agriculturists of the studied area and all cases of occupational diseases who were farmers visiting healthcare units for health services at the studied areas of the upper Northeast of Thailand, during the three years from 1 ^st^ January, 2014 to 31 ^st^ December, 2016. The inclusion criteria for farmers from the HDC were those registered under the occupational codes shown in Table 1. The registered agriculturists from a provincial agriculture office of Roi-Et and Udon Thani provinces who were included as the population in this study met the inclusion criteria of cultivated farming classifications according to agricultural activity during 2014-2016.

**Table 1.** Occupational code and meaning as the inclusion criteria for data from the Health Data Center (HDC) regarding services of agriculturists.

Occupational code	Meaning of occupational code
6111	Farm growers, farmers
6112	Fruit tree or rubber planters, rubber growers
6113	Horticultural growers, ornamental flower planters, planters in a nursery, ornamental plant growers, gardeners, mushroom growers
6114	Mixed croppers
9211	Fruit collectors, farmers/vegetable growers
9213	Crop workers with animal husbandry
9214	Horticultural and ornamental planters, field mowers, planters
7544	Sprayers for pest control and insect control

For analysis of the morbidity rate of occupational diseases among agriculturists in the upper northeast region, this study used two provinces as representative of the upper northeast of Thailand. The agriculturists from Roi-Et province were representative of PH region 7 and those of Udon Thani province were representative of PH region 8 of Thailand, to compare the healthcare service visits for all cases among farmers in provinces, or PH regions, to those of Thailand as a whole. All registered agriculturists of the two provinces were the representative population of agriculturists in those provinces in Thailand as the nature of the impact from agricultural work was found to be similar across from other areas of Thailand as Roi-Et is more real agriculture among these two provinces. ^31^^,^^32^

### Data collection

HDC data collection

The health database reports for national and PH region 7 and PH region 8 levels in the northeast region of Thailand were collected by using the ICD-10 code of occupational disease used in reports to the HDC ^22^^,^^33^^–^^37^ over 2014-2016. The data collected were the total number of planting farmers according to occupation code ( Table 1) and the number of cases of occupational disease for five occupational diseases classified by ICD-10 ^22^^,^^33^^–^^37^ ( Table 2). The total number of registered farmers to the Ministry of Agriculture and Cooperatives and the provincial agriculture office was collected from agricultural database. ^31^ The inclusion criteria for farmers from the HDC were those registered under the occupational codes shown in Table 1.

**Table 2.** Inclusion and exclusion criteria for data from ICD-10 codes in occupational disease as identified by the HDC service reports for ICD-10 and the 43 health files obtained from the provincial public health office.

Occupational disease	ICD-10 codes	Notes *
Toxicity from pesticides (pesticide toxicity)	T600, T601, T602, T603, T604, T608, T609	Exclude self-harm or suicide (external code of ICD-10: X68)
Chronic lower respiratory tract disease (lung diseases)	J40-J47	Considered health service visitors with the age group of 15-59 years old of the HDC database and the registered farmers (from provincial agriculture office) of the 43 health files
Work-related injuries (injuries)	S00–S99, T00-T35	^+^Considered with external code where the 5 ^th^ digit (activities) was 2 (work in a career) or missing
Hearing loss from noise and heat-related disease (noise and heat diseases)	H833, H903-H905, T670-T679	
Work-related musculoskeletal disorders (WMSDs)	M00-M99, G560	^+^Considered with additional code Y96 (occupation) or missing

* additional code consideration or exclusion is correlated with the definition of work-related diseases or injuries.

^+^ In case of missing codes of the 5 ^th^ digit (activities, 2) and Y96, they were also countable for the ICD-10 cases from main code consideration of occupational diseases or injuries among registered farmers from provincial agriculture office.

Provincial agriculture offices of Roi-Et and Udon Thani provinces data collection

Those data of the ICD-10 code and the registered agriculturists were also collected as a big dataset for Roi-Et and Udon Thani provinces as representative provinces in the upper northeast regions of Thailand. The secondary data was collected from a provincial agriculture office was as a big dataset with the number and lists of registered agriculturists in the pilot study areas (Roi-Et and Udon provinces) during 2014-2016. The record was explored and copied into an excel file by the information technology personnel who responsible for database of a provincial agriculture office with permission for the use of the data in this study. Reports were included containing the name, gender, house address, farming activities, and planting areas of all registered farmers during 2014-2016 from the Roi-Et and Udon Thani provinces who were cultivated farmers classified according to agricultural activity, i.e., cultivating rice, cassava, corn, soybean, or sugarcane as in the report Form 04-2.

HIS database data collection

Another big dataset collected from the provincial public health offices was the ICD-10 code record of the health standard data structure (43 health files of file 10, file 15, and file 19) on cases visiting Primary Healthcare Units (PCUs) and the secondary or tertiary hospitals in Roi-Et and Udon provinces for healthcare services in 2014 to 2016). Health-related information of registered agriculturists regarding cases with ICD-10 codes, was listed in the 43 health files of the health standards data structure and standards used for the hospital information systems (HIS) of the hospitals in Roi-Et and Udon Thani provinces: file 10 – diagnosis (opd), file 15 – diagnosis (ipd), and file 19 – surveillance. ICD-10 codes related to occupational disease and the disease data in an excel file was collated using the inclusion and exclusion criteria outlined in Table 2. Those ICD-10 codes were explored and copied from provincial health database by the permission code available only for the information technology personnel of the organization. Raw dataset collected under permission access and restriction use of ICD-10 codes and lists of registered farmers were collated and sorted by the installed hardware to computer including: Processor of CPU @ 1.99 GHz, Installed RAM of 16.0 GB, System type of 64-bit operating system, x64-based processor, and Software including Windows 10 or equal to higher version and Microsoft office (excel program) 2016 licensed by Khon Kaen University or equal to higher version. Duplicated registration of farmers or disease accounts were screened out and removed for duplication and showed that the final number of registered farmers in the period 2014 to 2016 of the Udon Thani province was 154,478, while that of Roi-Et was 207,465 farmers. The ICD-10 codes met the inclusion and exclusion criteria outlined as shown in Table 2 for occupational disease and injuries were considered for analysis of occupational diseases among the registered farmers. The collaborated data of registered farmer and accounted ICD-10 codes among the registered was explored and used for the morbidity rate analysis.

### Data analysis

STATA version 10.0 software ( https://www.stata.com/) (StataCorp LLC: College Station, TX) was used for all statistical analyses in this study. A freely accessible alternative software which can be used to complete the statistical analyses in this study would be program R for statistical computing ( https://www.r-project.org/). Categorical data were presented as numbers and percentages. To estimate the annual morbidity rate of an occupational disease with a confidence interval (95% confidence interval) among registered agriculturists from 2014-2016. The data of occupational diseases among planting farmers or registered agriculturists was analysed for morbidity rate as per the following formula:

$$\text{Morbidity rate}=\frac{\text{Number of cases reported among registered agriculturists at an area of interest}}{\text{Total number of farmers (registered agriculturists) at an area of interest}}\times 100,000$$ (1)

In case of missing codes of the 5 ^th^ digit (activities, 2) for work-related injuries or Y96 for work-related musculoskeletal disorders (WMSDs), they were also countable for the ICD-10 cases of occupational diseases or injuries among registered farmers from provincial agriculture office by considering the main ICD-10 code.

The underlying data (TABLE A B C: Situations of work-related diseases and injuries among agriculturists in the upper northeast regions of Thailand) of this study is available in the underlying data statement. ^46^

## Results

In 2014-2016, the number of registered farmers collected from provincial agriculture office in Roi-Et and Udon Thani provinces was 207,465 and 154,478, respectively. There were 53,794 (34.82%) total cases who visited for health services in Udon Thani, and 77,438 (37.32%) total cases in Roi-Et during the three-year period. The number of visiting cases in each year varies upon types of diseases and, among those health problems, we found the highest cases from injuries both in Roi-Et (n=20,341) and Udon-Thani (n=14,025) provinces in 2016.

**Figure 1.**
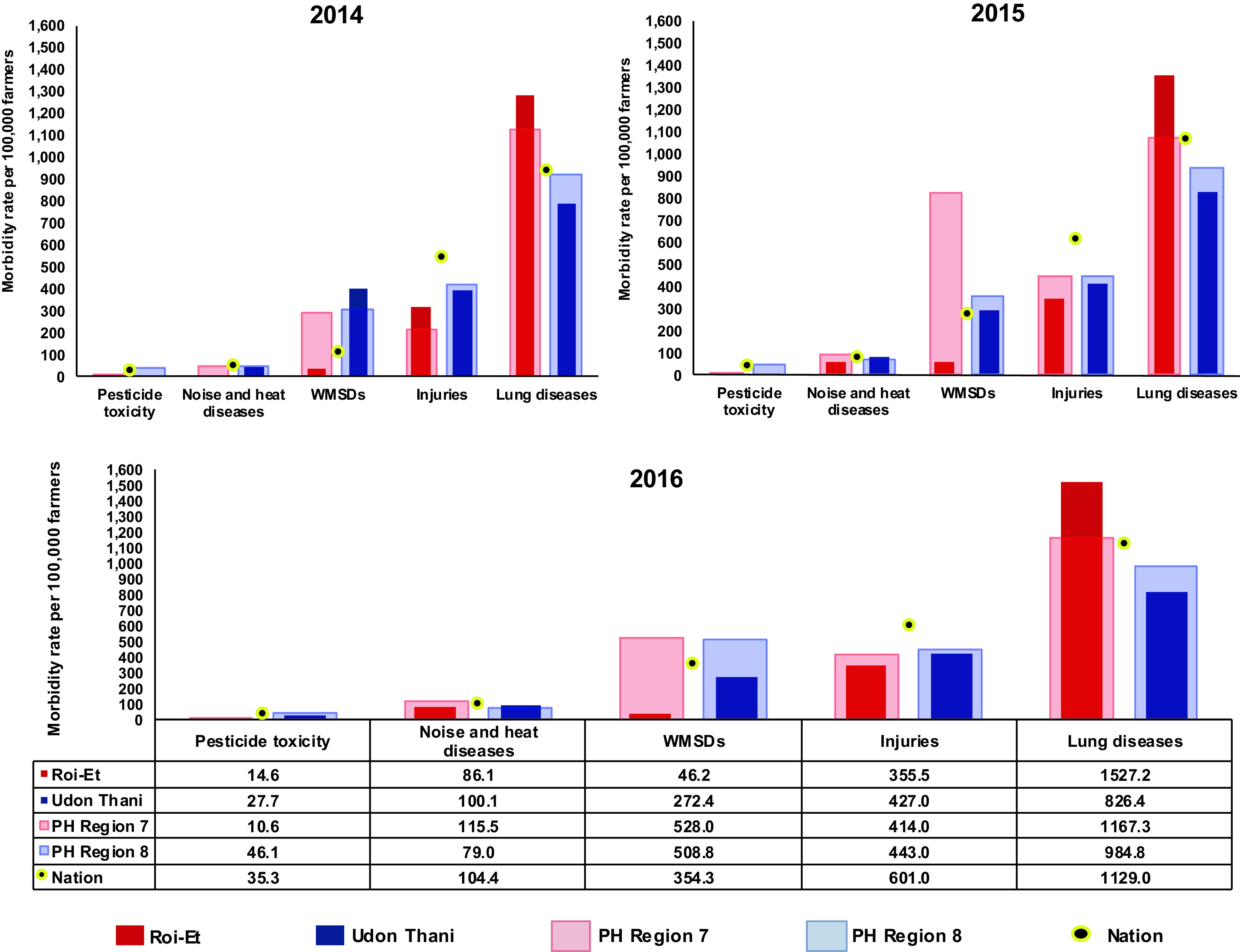
Morbidity rate per 100,000 farmers, classified according to occupational diseases and injuries recorded by the Health Data Center (HDC) of Thailand from 2014 to 2016.

For the number of farmers from HDC database, the number of HDC farmers varied upon types of diseases or injuries as well as the number of cases of diseases as shown in Table 3. There were the highest number of farmers accessed to healthcare from cases of noise and heat-related disease in Roi-Et (82,458) and Udon Thani (1,324,168). The number of registered farmers recorded of Ministry of Agriculture and Cooperatives ^31^ were 207,780 and 163,704 for Roi-Et and Udon Thani in 2016, respectively. The morbidity rate of occupational disease in Thailand, i.e., WMSDs, noise- and heat-related diseases, and pesticide toxicity (per 100,000 farmers), increased between 2014 and 2016, according to the HDC database. According to national results, the results of PH region 8 and Udon Thani supported this trend of increasing morbidity rate (as shown in Figure 1). Noise- and heat-related injuries have been increasing every year in all regions, which is also supported by the rates from Roi-Et province and PH region 7.

**Table 3.** Health Data Centre (HDC) morbidity rates (per 100,000 farmers) of occupational diseases and injuries, and comparison of the number of farmers from the HDC database and the number of registered farmers in 2016.

Occupational disease	Area/region	Number of cases	Number of HDC farmers ^†^	Number of registered farmers ^‡^	Morbidity rate per 100,000 HDC farmers *
WMSDs	Nation	41,912	11,829,540	4,178,064	354.30
	Roi-Et	19	41,108	207,780	46.22
	Udon Thani	1,800	660,883	163,704	272.36
Noise- and heat-related diseases	Nation	25,370	24,293,821	4,178,064	104.43
	Roi-Et	71	82,458	207,780	86.10
	Udon Thani	1,326	1,324,168	163,704	100.14
Pesticide toxicity	Nation	4,293	12,157,336	4,178,064	35.31
	Roi-Et	6	40,993	207,780	14.64
	Udon Thani	184	663,389	163,704	27.74
Injuries	Nation	75,612	12,580,864	4,178,064	601.01
	Roi-Et	147	41,350	207,780	355.50
	Udon Thani	2,837	663,729	163,704	427.00
Lung diseases **	Nation	-	-	4,178,064	-
	Roi-Et	-	-	207,780	-
	Udon Thani	-	-	163,704	-

^†^ the number of planting farmers as per the occupational code from the Health Data Centre (HDC) database.

^‡^ the number of registered farmers of the Ministry of Agriculture and Cooperatives. ^36^

* HDC morbidity rate per 100,000 HDC farmers.

** There was no report available of occupational disease category by HDC.

### Morbidity rate of work-related disease and injuries among farmers who visited healthcare service providers

This study analyzed the rate of chronic lower respiratory symptoms (J40-J47) as one category of lung diseases among farmers between 2014 and 2016. This was analysed in this manner following the categories of the top five prevalence of occupational diseases among crop planting farmers ^23^ as in Table 2. The disease was not directly reported as representative of a kind of occupational disease classified in the HDC database, but there was a reported data available as categories by age groups for 5-9 years, 10-14 years, 15-59 years, and 60 years or more, which could be accountable for the cases of workforce ages as agriculturists. ^22^ In 2016, the national rate of lung disease ranged from 943.9-1,129.0 per 100,000 farmers, and the highest rate was found in Roi-Et (1,527.2), which was higher than that of the national total. The rate of lung disease was followed in order by the rates of injuries, WMSDs, noise- and heat-related diseases, and pesticide toxicity, respectively, and there was a higher morbidity rate in Udon Thani compared to Roi-Et. In Roi-Et, injuries were found to be most prevalent at 319.4 per 100,000 farmers in 2014, which was followed by WMSDs (41.0), pesticide toxicity (11.4), and noise- and heat-related diseases (6.8), while the top-ranking rate of Udon Thani was that of WMSDs (405.3), which was followed by those of noise- and heat-related diseases (47.4), and pesticide toxicity (12.8), while the injuries rate was 400.0. In 2015, apart from injuries (352.2), lung disease (1359.3) was the top-ranking rate of diseases among farmers in Roi-Et, the following morbidity rate of diseases were noise- and heat-related diseases (67.3), WMSDs (62.7), and pesticide toxicity (14.5). Meanwhile, in Udon Thani, the morbidity rate of lung disease was the highest rate (831.6), followed by WMSDs (296.8), noise-and heat-related diseases (88.0). A similar trend was found during 2014-2016, in that the morbidity rates of Udon Thani, or PH region 8, were mostly representative of the national rate. In 2016, the morbidity rate of chronic lower respiratory symptoms (lung diseases) was the highest among all diseases and injuries in Roi-Et Province and was also shown to be the highest among all diseases and injuries when compared to other regions and the nation ( Figure 1, see underlying data: TABLE A). ^46^

Even though the number of cases of diseases was stable, the number of people who had a recorded occupation of farmer in the HDC database and the number of registered farmers were different. Udon Thani as well as the nation always had a higher recorded number of farmers in comparison to the registered number of farmers, which contrasted with Roi-Et, as shown in Table 3.

In 2016, all rates of Udon Thani were higher than those of Roi-Et, i.e., with a 5.89, 1.16, 1.89, and 1.20-times higher rate of WMSDs, noise- and heat-related diseases, pesticide toxicity, and injuries, respectively.

Although the number of registered farmers of Roi-Et Province was higher than that of Udon Thani, the recorded cases or number of farmers to health service visits were lower compared to Udon Thani, for all diseases and injuries. The morbidity rate of disease was higher in Udon Thani in comparison to Roi-Et. It was found that the morbidity rates of the nation and Udon Thani Province had been increasing similarly and were much higher than the morbidity rate of Roi-Et.

When comparing the rate of occupational disease and injuries among registered farmers in 2016 to the rate of farmers who visited health service providers, the higher rate was found in the registered farmers for WMSDs, noise- and heat-related diseases, and injuries (pesticide toxicity was scarcely different), especially in Roi-Et, where the rate was 27.58 times higher for injuries and 16.32 times higher for WMSDs. Regarding those who identified as farmers when visiting Udon Thani health service providers, they had a 1.76 times lower rate of WMSDs and a 15.83 times lower rate of injuries than registered farmers. Similarly, the rates of noise- and heat-related diseases among those who identified as farmers in Udon Thani and Roi-Et provinces were about two times lower in the HDC database, in comparison to the rates of registered farmers.

### Morbidity rate of work-related disease and injuries of registered farmers visited health care providers in 2016

According to the secondary dataset of the health standard data structure (43 health files) and the number of agriculturists registered to the provincial agricultural office during 2014-2016, without duplicated registration of farmers or disease accounts, the analysis showed the number of registered farmers in 2014 to 2016 of Udon Thani Province was 154,478, while that of Roi-Et was 207,465 farmers (see underlying data: TABLE C). ^46^ By utilizing the 43 health files data of the ICD-10 code of occupational cases during 2014 to 2016, there were 53,794 total cases of registered farmers accessed for health services in Udon Thani, and 77,438 total cases in Roi-Et. The number of cases among registered agriculturist varied upon types of diseases or injuries. The results of morbidity rates in 2016 are presented in Table 4. The ranking of morbidity rates of work-related disease among registered farmers was as follows: WMSDs, noise- and heat-related diseases, and pesticide toxicity, respectively. Work-related injuries had the highest rate among all disorders of Udon Thani and Roi-Et ( Table 4).

**Table 4.** Morbidity rate (per 100,000 farmers) of occupational diseases and injuries among registered farmers of the provincial agriculture office in 2016 utilized from 43 health files data from the provincial public health office.

Diseases/injuries	Cases		Morbidity rate (95% CI)	
	Roi-Et	Udon Thani	Roi-Et	Udon Thani
WMSDs	1,565	740	754.3 (717.6-792.5)	479.1 (604.2-684.6)
Noise- and heat-related diseases	460	321	221.7 (201.9-242.9)	207.7 (203.3-306.6)
Pesticide toxicity	18	41	8.7 (5.1-13.7)	26.5 (16.8-46.3)
Injuries	20,341	14,025	9,804.6 (9,676.9-9,933.3)	9,078.9 (8,936.1-9,223.3)
Chronic lower respiratory tract disease (Lung diseases)	5,771	3,520	2,781.7 (2,711.3 – 2,853.3)	2,278.6 (2,204.8 – 2,354.3)

### The trend of work-related disease among registered farmers

**Figure 2.**
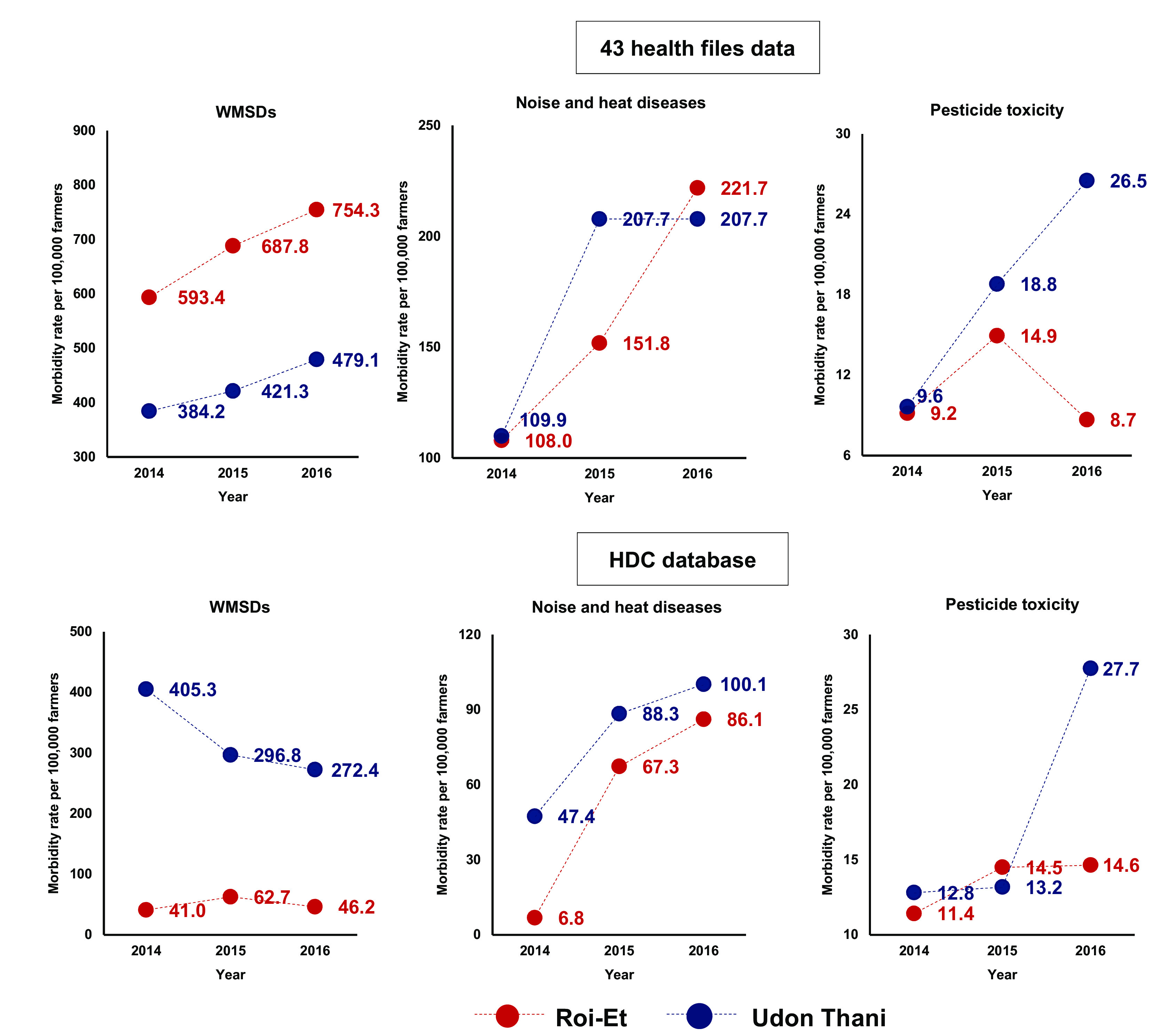
Morbidity rate per 100,000 farmers in 2014-2016, classified by occupational disease, from the 43 health files data from the agricultural registry and the Health Data Centre (HDC) database.

The significance of diseases among registered farmers changed, in that the rate of WMSDs in Roi-Et became higher than that of Udon Thani, according to the 43 health files data structure, when compared to the HDC database ( Figure 2 and underlying data: TABLE B, C). ^46^

From the 43 health files data structure, it was found that the morbidity rates among the registered farmers of Roi-Et were highest ranked as follows: WMSDs, noise- and heat-related diseases, and pesticide toxicity, respectively, in every year from 2014 to 2016. The morbidity rate of registered farmers was not consistent with those who identified as farmers, as recorded by the HDC, in every year from 2014 to 2016. In 2016, farmers in Roi-Et had a higher morbidity rate of WMSDs, and the rate of noise- and heat-related diseases was higher than that of Udon Thani. The rates and trends increased every year in this manner, except for pesticide toxicity, which had a lower rate in Roi-Et when compared to that of Udon Thani. In Udon Thani, the morbidity rates from the 43 health files were found to have the same ranking as Roi-Et for every disease. WMSDs had the highest morbidity rate, followed by noise- and heat-related diseases and pesticide toxicity, respectively, and all those rates had increased in the same manner from 2014 to 2016.

The results from the HDC showed that of all occupational diseases, there was a clear increasing trend for pesticide toxicity and noise- and heat-related diseases, which was not consistent with the decreasing rate of WMSDs in Udon Thani. However, Udon Thani had a higher overall rate than Roi-Et for all occupational diseases.

## Discussion

The numbers of work-related diseases and injuries reported at present among Thai farmers are likely to be lower than the true numbers because the health surveillance among farmers or agricultural workers may not be correct, as can be seen from the following findings and discussion.

By using registered farmers as the population for the morbidity rate calculation, which was different from using those who used a farmer occupational code as the population to calculate the rate from the HDC database, one could explain the inaccurate number of agriculturists found in healthcare service reports. The number of farmers from the HDC database may sometimes include duplicate recordings of farmers who visited healthcare providers more than once or sometimes be missing the occupational code identification of an agriculturist. That inaccurate number of farmers from the HDC database has led to incorrect results of morbidity rate. Therefore, for the next step of analysis, it was necessary to study registered agriculturists for accounted cases of occupational disease among agriculturists.

Those results of work-related diseases and injuries, excluding lung disease, among registered agriculturists, from the two case studies of Udon Thani and Roi-Et provinces from analysis of the HDC database and the 43 health files data, showed the same ranking of morbidity rate, which were injuries, WMSDs, noise- and heat-related diseases, and pesticide toxicity, respectively. Consequently, both Udon Thani and Roi-Et were possibly representative of national rates. For the case of lung diseases, the number of cases who had lung disease specific to the age group of between 15 to 59 years of age was analysis for the morbidity rate from HDC database, the disease was not further reported in the HDC database as occupational disease as mentioned before. It showed the highest morbidity rate from our analysis for chronic lower respiratory tract disease, or a specified lung disease among occupational diseases. However, when considering the morbidity rates (per 100,000 registers farmers) of diseases and injuries from 2014 to 2016 found in 43 health files data, it was found that the highest morbidity rate from our analysis was not for a specified lung disease which had not been reported before as an occupational disease in the disease surveillance system of the Ministry of Public Health, Thailand. The high morbidity rate of occupational lung disease, which was not yet covered by the HDC database, might be explained by the fact that in disease diagnosis, it was not found to have an external cause, which would require recoding to Y96 to specify an occupational disease group. Some other diseases related to exposure to agrochemical hazards were recommended from the previous studies, e.g., Parkinson's disease ^38^; cancers of specific organs, e.g., lung, pancreas, lymphohematopoietic organs, bladder, prostate gland, ^39^ or thyroid ^40^; gestational diabetes mellitus (GDM) ^41^ or diabetes ^42^; etc. In covering those diseases to build up the surveillance system among agriculturists, some more investigation is still needed to confirm an association between each disease and a specific toxic substance used in pest control. Moreover, occupational lung disease should be considered in health surveillance of disease among farmers as per the previous report done by using a systematic review among Thai agriculturists. ^43^

Excluding lung disease, the three top-ranking rates across all the regions and years were injuries and/or WMSDs, followed by hearing loss and diseases from heat, and pesticide toxicity, respectively. In addition, the rate of injuries was highest among all occupational diseases in Roi-Et, Udon Thani, and the nation. Udon Thani, which was chosen as a good representative province of PH Region 8, had a top-ranking rate which was the same as the nation, namely that of injuries, followed by WMSDs, noise- and heat-related diseases, and pesticide toxicity, respectively. These findings confirmed the similar results found among registered crop farmers in Nongbualamphu, one of the provinces in PH Region 8, Thailand, ^23^ as well as those found in other countries in Asia, ^2^ Canada, ^3^ Europe, ^4^^,^^6^ and America. ^5^^,^^7^

In cases of toxicity from pesticides in 2016, the morbidity rate in Roi-Et Province was lower than previously reported among southern Roi-Et farmers during the same period, ^26^ which could be explained by local agricultural behaviors and the fact that pesticide toxicity report cases depended on the disease surveillance of each regional public health service. ^44^ Moreover, it was noteworthy that Roi-Et provincial-level case reports were very low in number compared to those of Udon Thani ( Table 3).

According to the analysis of ICD-10 utilized from the 43 health files standard among registered farmers, the morbidity rate of WMSDs in Roi-Et was around double that of Udon Thani. In contrast, the morbidity rate of WMSDs in Udon Thani, according to the HDC database, was around 10 times higher than that of Roi-Et. Regarding this comparison of morbidity rates of WMSDs, namely the HDC database versus the 43 health files data records, the trends were found to be unclear. This might be explained by the fact that case reports of WMSDs are not only specific to farming work, or they have multi-factor causes. ^43^ Another explanation might be the wrong or missing diagnostic coding of Y96, which signifies a specified occupational cause, which was confirmed by our findings to support the previous study. ^44^

Udon Thani had a higher rate than Roi-Et with regard to noise- and heat-related diseases and pesticide toxicity, according to HDC database analysis as well as analysis of the 43 health files of the standard data structure. Increasing rates were observed from 2014-2016 among Udon Thani and Roi-Et agriculturists. These two occupational disease groups were confirmed as the major health problems among agriculturists in Thailand. ^43^

Infectious diseases, such as leptospirosis and melioidosis, had the highest prevalence in farmers, but there was no infectious disease group shown for occupational disease in the HDC database. This was not only the case in Thailand; a previous study in Poland showed a significantly higher rate in farmers who had been exposed to repeated tick bites, ^45^ which corresponded to annual reports in Greece from 2004–2015. ^15^ Moreover, brucellosis cases have been found in farmers and livestock breeders, with a high incidence rate of 7.1 per 100,000 population. ^15^

From the HDC database, it was found that the top three morbidity rates in Roi-Et, in 2014, were the morbidity rate of WMSDs followed by those of pesticide toxicity and noise- and heat-related diseases, respectively. However, the morbidity rates during 2015 and 2016 changed in order. Meanwhile, the top three morbidity rates of Udon Thani (2014-2016) were the morbidity rate of WMSDs followed those of by noise- and heat-related diseases and pesticide toxicity, respectively. That explains why Roi-Et was unable to be provide good representative data because the province had not reported data as forecast, which contrasted with Udon Thani Province in this study. The morbidity rates from the health data of Roi-Et province showed congruous results among the registered farmers. The highest rate was for work-related injuries, followed by WMSDs, hearing loss and heat-related disease, and pesticide toxicity, respectively. Similar results were found in Udon Thani, where rates of work-related injuries, WMSDs, and noise- and heat-related disease were similar and close to the national rates. Hence, these results reveal that the provincial data of Udon Thani can be representative of the nation. The consistency of the database is shown by the top three highest morbidity rates of disease from the previous study in crop farmers of Nongbualamphu province, which were shown to be WMSDs, noise- and heat-related disease, followed by skin irritation. ^23^ Moreover, the number of reported cases was very different between Udon Thani and Roi-Et, and the morbidity rate of pesticide toxicity in Roi-Et was dramatically lower than that of the previous report from 2016-2018 among registered farmers in Sakon Nakhon, a province in PH Region 8. ^25^ Those cases mentioning skin disease were suspect due to a combined effect in the group of pesticide toxicity, which is that of a reported acute symptom (irritant), and confirmed the previous studies. ^25^^,^^26^

These disputations with strong evidence suggest health surveillance of occupational disease among agriculture workers (informal workers) or Thai farmers by 1) realizing the diagnosis with a coding record of Y96, 2) improving the recording method of occupation to be used practically for agriculturists, and 3) promoting agriculture workers to register on the health database. It was more than interesting that some have mentioned other diseases should be added to ICD-10 for occupational disease, i.e., lung disease, skin disease and infectious disease, for the occupational surveillance system.

## Conclusions

The morbidity-rate ranking of work-related diseases and injuries among agriculturists in the upper northeast of Thailand was WMSDs, noise- and heat-related disease, and pesticide toxicity, respectively. When comparing the morbidity rates of farmers from the 43 health files data to those of the HDC database, the closest rates were found in the pesticide toxicity rates of Roi-Et and Udon Thani, which contrasted with those of noise- and heat-related diseases. Moreover, in 2016, the rates of WMSDs in Roi-Et from the 43 health files data were more than 10 times higher than those from the HDC data, which was like those of noise- and heat-related diseases. All work-related diseases of the 43 health files data had increasing trends; however, data from the HDC showed rates of WMSDs were in a decreasing trend. Regarding each individual province, Roi-Et had an increasing trend for all diseases, for both data from the 43 health files data and data from the HDC, but a disease of Udon Thani had a clearly decreasing trend, which can be explained by the occupational coding that Udon Thani has developed, thus strongly communicating that WMSDs had not been caused by occupation or only by the agricultural activity of farmers. Database of work-related diseases and injuries among agriculturists in the northeast of Thailand do not reflect reality at the present. There are two major reasons why the morbidity rates are not accurate. Firstly, several cases were completed without identifying as ICD-10 of specifying with extra code Y96 as occupational causes. Secondly, the total number of the population of interest, which was agriculturists, was not the true number. Therefore, recording an occupation when visiting health service providers should be promoted for the future, and those agriculturists should be registered in a healthcare database or surveillance system.

## Data availability

### Underlying data

The underlying data for this study is secondary data collected by the Health Data Centre and agricultural registries and is restricted due to ethical and data protection considerations. Under the ethical approval from the Human Research Ethics Committee of Khon Kaen University (No. HE592154), participants’ data was not allowed to be shared and the secondary data of health dataset and registered farmer dataset were utilized under restriction. The individual information or further reused inclusion data is not permitted under the ethically approved conditions for human research. This was confirmed under the data protection of human/participant privacy as no individuals gave explicit written consent that their identifiable data can be made publicly available. For more information, this research had no personal identified data for accessible because of the ethical issue that does not allow us to hold the personal data of each participant and cannot be shared any data after closing research.

The raw dataset collected under permission access and restriction use of ICD-10 codes are the 43 health files lists of Roi-Et and Udon Thani provinces in the period 2014 to 2016: file 10 – diagnosis (opd), file 15 – diagnosis (ipd), and file 19 – surveillance and the lists of registered agriculturists in Roi-Et and Udon Thani provinces during 2014-2016. To request access to the underlying data, please email the primary investigator (Sunisa Chaiklieng; csunis@kku.ac.th as the corresponding author. Applications will be reviewed on a case-by-case basis.

The intermediary data that can be de-identified without compromising anonymity are all presented already in the results and underlying data (TABLE A, B, C) and listed below.

Open Science Framework (OSF): Situations of work-related diseases and injuries among agriculturists in the upper northeast regions of Thailand. https://doi.org/10.17605/OSF.IO/2JPZM. ^46^

The project contains the following underlying data:

• TABLE A B C. (Table 1 - data for Figure 1; Table B - data for Figure 2 that is related to HDC database; and Table C - data for Figure 2 that is related to 43 Health files)

Data are available under the terms of the Creative Commons Zero “No rights reserved” data waiver (CC0 1.0 Public domain dedication).
