# Situations of work-related diseases and injuries among agriculturists in the upper northeast regions of Thailand

**DOI:** 10.12688/f1000research.73221.2

**Published:** 2023-01-23

**Authors:** Sunisa Chaiklieng, Chuthamas Chagkornburee, Pornnapa Suggaravetsiri

**Affiliations:** 1 Department of Environmental Health, Occupational Health and Safety, Faculty of Public Health, Khon Kaen University,, Khon Kaen, 40002, Thailand; 2 Master of Science Program in Occupational Health and Safety, Faculty of Public Health, Faculty of Public Health, Khon Kaen University, Muang Khon Kaen, Khon Kaen, 40002, Thailand; 3 Department of Epidemiology and Biostatistics, Faculty of Public Health, Khon Kaen University, Khon Kaen, 40002, Thailand

**Keywords:** Big data, occupational disease, agriculturists, surveillance, ICD-10

## Abstract

**Background: **Agriculturists exposed to health hazards are affected by increased occupational disease. This retrospective study aimed to investigate situations of work-related diseases and injuries among agriculturists in upper northeast Thailand.

**Methods: **The secondary data of international classification of diseases 10 ^th^ revision (ICD-10) case reports of occupational disease among farmers, from the database of the Health Data Center (HDC), were used. The registered farmers data was collected as a dataset from the provincial agricultural office and the data of ICD-10 code utilised from the hospital information system (HIS) of healthcare services in Udon Thani and Roi-Et provinces, which was extracted for cases of work-related diseases and injuries of registered agriculturists. The annual morbidity rate of occupational diseases was analysed and presented at a rate per 100,000 farmers.

**Results: **Among farmers in the HDC database, lung disease, which was not reported as occupational disease of the HDC database, was the highest ranking of all diseases, followed by work-related musculoskeletal disorders (WMSDs), noise- and heat-related diseases, and pesticide toxicity, respectively, while the injury rate was as high as that of WMSDs. Those morbidity rates of Roi-Et and Udon Thani provinces were representative of the morbidity ranking of diseases of the nation and had increasing trends from 2014 to 2016. The number of farmers in the HDC database did not consistently reflect the number of registered farmers in the agricultural database.

**Conclusions: **Situations of work-related diseases and injuries discovered among registered farmers reflect the health problems of Thai agriculturists, and the underestimation in the reported disease rate in the health database is explained by big data analysis, which showed that work-related cases with an identifying code of Y96 had rarely been reported among agriculturists. Therefore, Thai agriculturists should be supported in registration with occupational diseases and injuries surveillance as holistic healthcare

## Introduction

Agriculture is an essential source of livelihood for developing countries. However, it can lead to poor health and is linked to the main causes of health hazards of agriculturists affected by infection, injuries, chronic exposure, and increased occupational disease. ^1^ According to a global report on occupational diseases, the highest prevalence of such disease was work-related musculoskeletal disorders (WMSDs), which ranged between 55.6-97.2%. ^2^ ^–^ ^4^ The highest reported prevalence was in Korea (97.2%), ^2^ followed by Saskatchewan province of Canada (85.6%), ^3^ and Ireland (55.6%). ^4^ The next most prevalent disease was heat-related disease, which ranged from 35.6-72.3%. ^5^ ^–^ ^8^ The highest reported prevalence was found in Eastern North Carolina, USA, ^5^ followed by Northeast Italy, ^6^ and North Carolina, USA. ^7^ ^,^ ^8^ Additionally, prevalence of hearing loss from noise was reported at levels of 16.9-36.1%, ^9^ ^,^ ^10^ which were 16.9% ^9^ in Korea and 36.1% ^10^ in the USA, while work-related injuries were reported at a prevalence of 69.0% in Nepal. ^11^ Infectious disease was reported in ranges between 0.7-13.5%, ^12^ ^–^ ^14^ where brucellosis cases in Greek farmers and livestock breeders corresponded to an annual incidence rate of 7.1 per 100,000 population, ^15^ which were 8.9% in the Western Cape of South Africa. ^16^ The pesticide toxicity was reported as the rate of 8.8-17.0%, which were 8.8% in China, ^17^ and 17.0% in the Sunsari District of Nepal. ^18^ Chronic lower respiratory tract disease was also reported as chronic obstructive pulmonary disease (COPD) and was found in a systematic review to be from 3.0% to 68.0%, ^19^ as in France, where the rate of COPD was 9.5%, ^20^ and South Africa, where the rate was 3.0% for asthma. ^21^

From the survey results in 2019, it was found that the total number of employed persons in Thailand was 37.5 million persons. The number of workforce members who had no social security or were informal workers was about 20.4 million, or 54.3%. By region, those in informal employment working in the Northeast made up the largest proportion (34.9%), followed by Central Thailand (23.4%), the North (20.9%), and the South (14.0%). ^22^ When the economic activities of those in informal employment were considered, it was found that more than half of all informally employed persons worked in the agriculture sector (about 11.5 million, or 56.4%). ^22^

In Thailand, the Health Data Center (HDC) ( https://hdcservice.moph.go.th/hdc/main/index.php), which is the central health database system of Thailand, collects and analyses the disease surveillance system data of all healthcare services under the provincial public health office by using 43 files, or standard structured health data classified with ICD-10 codes for diseases and disorders, and records the patient’s information every time they receive services from officials. The health database of the HDC showed that the highest prevalence of disease between 2014 and 2016 was for work-related injuries (about 0.6%), followed by WMSDs (about 0.3%), noise-induced hearing loss and heat-related disease (0.2%), and pesticide toxicity (0.04%). There was no statistical report on work-related diseases which included respiratory symptoms or lung disease, and infectious disease on the health database of the HDC. In 2019, the prevalence of chronic lower respiratory tract disease was reported of 15-59 years old population to be 1.8%. ^23^

The previous retrospective study in 2016, which showed a higher morbidity rate of all cases in the research from the Nongbualamphu province of Thailand, found the highest prevalence was for WMSDs (21.7%), followed by heat- and pressure-related diseases (5.2%). ^24^ The pesticide toxicity was reported in the range of 0.14-0.18% of registered farmers, ^24^ ^,^ ^25^ which can be compared to the previous prevalence rate of 48.48 per 100,000 registered farmers in Southern Roi-Et. ^26^ The study also indicated a prevalence rate of infectious disease at 0.51%, ^24^ with chronic lower respiratory tract disease (1.11%) and skin disease (1.5%) ^24^ as additional diseases which occurred. The case-control study of registered agriculturists in Khon Kaen confirmed that the noise-induced hearing loss cases from the 43 health files data had been diagnosed through the audiometer test of those agriculturists. ^27^ Work-related injuries had a prevalence of 20.1% among the farmers of the Phayao province ^28^ and the three-year morbidity rate of heat-related illness in our most recent report (2020) was 13.5 per 100,000 farmers, or 0.014%, in registered agriculturists of Khon Kaen province. ^29^

Those work-related diseases and injuries in Thailand were identified by the International Statistical Classification of Diseases and Related Health Problems 10 ^th^ revision (ICD-10) ( https://icd.who.int/browse10/2019/en) ^30^ and the specified Y96 external code (work related condition). Nowadays, however, some cases of agriculturists are not practically identified, and this leads to inconsistency in data as above. Hence, healthcare for agricultural workers in Thailand is not covered by labour legislation and as a result they only access services from the National Health Security Office (NHSO) ( https://eng.nhso.go.th/view/1/Home/EN-US), ^31^ without claiming compensation for diseases related to agricultural activities. Although occupational disease case reports are important for enabling the surveillance system to carry out national health policy among agriculturists, there is still no representative morbidity rate of the upper northeast of Thailand. Therefore, this study aimed to investigate situations of work-related diseases and injuries among agriculturists in the upper northeast of Thailand by using the case study of the Udon Thani and Roi-Et provinces.

## Methods

### Ethical approval

This study was approved by the Human Research Ethics Committee of Khon Kaen University (No. HE592154). The underlying data for this study is secondary data collected by the Health Data Centre and agricultural registries and is restricted due to ethical and data protection considerations. Under the ethical approval from the Human Research Ethics Committee of Khon Kaen University (No. HE592154), participants’ data was not allowed to be shared and the secondary data of health dataset and registered farmer dataset were utilized under restriction. The individual information or further reused inclusion data is not permitted under the ethically approved conditions for human research. This was confirmed under the data protection of human/participant privacy as no individuals gave explicit written consent that their identifiable data can be made publicly available.

### Study design

This study was a retrospective analytic study that used secondary data from three sources, which were 1) The health database of the Health Data Center (HDC) of Thailand, between 2014 and 2016, for the number of cases of occupational disease among agriculturists (by occupational code) who visited public healthcare service providers as details in HDC data collection and public health region seven (PH region 7) and public health region eight (PH region 8), and Roi-Et and Udon Thani provinces in the upper northeast regions of Thailand; 2) The secondary dataset of the list of cultivating farmers who had registered with the provincial agriculture offices of Roi-Et ( http://www.roiet.doae.go.th/) and Udon Thani provinces ( http://www.udonthani.doae.go.th/) between 2014 and 2016 as in the report Form 04-2; and 3) The secondary data of ICD-10 utilised from the hospital information system (HIS) database as detailed in the HIS data collection that used for routine care services in primary care units, secondary, and tertiary hospitals was selected from the provinces of the pilot study areas (Roi-Et and Udon Thani).

### Population and sample size

The sample size of the retrospective descriptive study was the total number of registered agriculturists of the studied area and all cases of occupational diseases who were farmers visiting healthcare units for health services at the studied areas of the upper Northeast of Thailand, during the three years from 1 ^st^ January, 2014 to 31 ^st^ December, 2016. The inclusion criteria for farmers from the HDC were those registered under the occupational codes shown in Table 1. The registered agriculturists from a provincial agriculture office of Roi-Et and Udon Thani provinces who were included as the population in this study met the inclusion criteria of cultivated farming classifications according to agricultural activity during 2014-2016.

**Table 1.** Occupational code and meaning as the inclusion criteria for data from the Health Data Center (HDC) regarding services of agriculturists.

Occupational code	Meaning of occupational code
6111	Farm growers, farmers
6112	Fruit tree or rubber planters, rubber growers
6113	Horticultural growers, ornamental flower planters, planters in a nursery, ornamental plant growers, gardeners, mushroom growers
6114	Mixed croppers
9211	Fruit collectors, farmers/vegetable growers
9213	Crop workers with animal husbandry
9214	Horticultural and ornamental planters, field mowers, planters
7544	Sprayers for pest control and insect control

For analysis of the morbidity rate of occupational diseases among agriculturists in the upper northeast region, this study used two provinces as representative of the upper northeast of Thailand. The agriculturists from Roi-Et province were representative of PH region 7 and those of Udon Thani province were representative of PH region 8 of Thailand; they were chosen to represent the healthcare service visits for all cases among farmers in provinces of the northeast of Thailand and be compared to those of Thailand as a whole. All registered agriculturists of the two provinces were the representative population of agriculturists in those provinces in Thailand as the nature of the impact from agricultural work was found to be similar across from other areas of Thailand. ^32^

### Data collection

#### HDC data collection

The health database reports for national and PH region 7 and PH region 8 levels in the northeast region of Thailand were collected by using the ICD-10 code of occupational disease used in reports to the HDC ^23^ ^,^ ^33^ ^–^ ^37^ from 2014 to 2016. The data collected were the total number of planting farmers according to occupation code ( Table 1) who had accessed healthcare in the area of interest and the number of cases of occupational disease among farmers who met the inclusion criteria for planting farmers reported to the HDC, with regard to four occupational diseases and injuries classified by icd-10 ^23^ ^,^ ^24^ ^,^ ^33^ ^–^ ^37^ ( Table 2). The total number of farmers registered nationwide with the Ministry of Agriculture and Cooperatives and provincial agriculture offices was collected from the agricultural database. ^32^

**Table 2.** Inclusion and exclusion criteria for data from ICD-10 codes in occupational disease as identified by the HDC service reports for ICD-10 and the 43 health files obtained from the provincial public health office.

Occupational disease	ICD-10 codes	Notes *
Toxicity from pesticides (pesticide toxicity)	T600, T601, T602, T603, T604, T608, T609	Exclude self-harm or suicide (external code of ICD-10: X68)
Chronic lower respiratory tract disease (lung diseases)	J40-J47	Considered health service visitors with the age group of 15-59 years old of the HDC database and the registered farmers (from provincial agriculture office) of the 43 health files
Work-related injuries (injuries)	S00–S99, T00-T35	^+^Considered with external code where the 5 ^th^ digit (activities) was 2 (work in a career) or missing
Hearing loss from noise and heat-related disease (noise and heat diseases)	H833, H903-H905, T670-T679	
Work-related musculoskeletal disorders (WMSDs)	M00-M99, G560	^+^Considered with additional code Y96 (occupation) or missing

^*^ Additional code consideration or exclusion is correlated with the definition of work-related diseases or injuries.

^+^ In case of missing codes of the 5 ^th^ digit (activities, 2) and Y96, they were also countable for the ICD-10 cases from main code consideration of occupational diseases or injuries among registered farmers from provincial agriculture office.

***Provincial agriculture offices of Roi-Et and Udon Thani provinces data collection***

Those data of the ICD-10 code and the registered agriculturists were also collected as a big dataset for Roi-Et and Udon Thani provinces as representative provinces in the upper northeast regions of Thailand. The secondary data that was collected from a provincial agriculture office was a big dataset with the number and lists of registered agriculturists in the pilot study areas (Roi-Et and Udon provinces) during 2014-2016. The record was explored and copied into an excel file by the information technology personnel who were responsible for the database of a provincial agriculture office with permission for the use of the data in this study. Reports included the identification number, name, gender, house address, farming activities, and planting areas of all registered farmers during 2014-2016 from the Roi-Et and Udon Thani provinces who were cultivating farmers classified according to agricultural activity, i.e., cultivating rice, cassava, corn, soybean, or sugarcane as in the report Form 04-2.

#### HIS database or 43 health files data collection

Another big dataset collected from the provincial public health offices was the ICD-10 code record of the health standard data structure (43 health files of file 10, file 15, and file 19) on cases visiting Primary Healthcare Units (PCUs) and the secondary or tertiary hospitals in Roi-Et and Udon provinces for healthcare services in 2014 to 2016). Health-related information of registered agriculturists regarding cases with ICD-10 codes, was listed in the 43 health files of the health standards data structure and standards used for the hospital information systems (HIS) of the hospitals in Roi-Et and Udon Thani provinces: file 10 – diagnosis (opd), file 15 – diagnosis (ipd), and file 19 – surveillance. The dataset of ICD-10 codes related to occupational disease and injuries data in an excel file or an SQL file were explored and copied from the provincial health database by using the permission code available only to the information technology personnel of the organisation. Data of ICD-10 code with access permission and restricted use was extracted from the 43 health files data based on identifiers of individuals registered as agriculturists with the Provincial Agriculture Office of Roi-Et or Udon Thani and sorted by using the hardware and installed software of the computer, which were a CPU @ 1.99 GHz, installed RAM of 16.0 GB, system type of a 64-bit operating system, x64-based processor, and software including Windows 10 or equal to a higher version and Microsoft Office (Excel program) 2016 licensed by Khon Kaen University, or equal to a higher version. The data was screened for duplicate registration of farmers or disease accounts, and they were removed for duplication; it was shown that the final number of registered agriculturists in the period of 2014 to 2016 in Udon Thani province was 154,478, while that in Roi-Et was 207,465 farmers. The ICD-10 codes met the inclusion and exclusion criteria outlined as shown in Table 2 for occupational disease and injuries were considered for analysis of occupational diseases among the registered agriculturists. The collaborated data of registered farmer and accounted ICD-10 codes among the registered was explored and used for the morbidity rate analysis per 100,000 registered farmers.

### Data analysis

STATA version 10.0 software ( https://www.stata.com/) (StataCorp LLC: College Station, TX) was used for all statistical analyses in this study. A freely accessible alternative software which can be used to complete the statistical analyses in this study would be program R for statistical computing ( https://www.r-project.org/). Categorical data were presented as numbers and percentages. To estimate the annual morbidity rate of an occupational disease with a confidence interval (95% confidence interval) among registered agriculturists from 2014-2016. The data of occupational diseases among planting farmers or registered agriculturists was analysed for morbidity rate as per the following formula: ^24^

$$\text{Morbidity rate}=\frac{\text{Number of cases reported among farmers or registered agriculturists}\;\text{in an area of interest}}{\text{Total number of farmers or registered agriculturists in an area of interest}}\times 100,000$$ (1)

In case of missing codes of the 5 ^th^ digit (activities, 2) for work-related injuries or Y96 for work-related musculoskeletal disorders (WMSDs), they were also countable for the ICD-10 cases of occupational diseases or injuries among registered farmers from provincial agriculture office by considering the main ICD-10 code.

The underlying data (TABLE A B C D: Situations of work-related diseases and injuries among agriculturists in the upper northeast regions of Thailand) of this study is available in the underlying data statement. ^49^

## Results

In 2014-2016, the number of registered farmers collected from the provincial agriculture office in Roi-Et and Udon Thani provinces was 207,465 and 154,478, respectively. Among those registered farmers, there were 53,794 (34.82%) total cases who visited for health services in Udon Thani, and 77,438 (37.32%) total cases in Roi-Et during 2014-2016.

For the number of farmers from HDC database, the number of HDC farmers varied upon types of diseases or injuries as well as the number of cases of diseases as shown in Table 3. The highest number of farmers accessed healthcare due to cases of noise and heat-related disease in Roi-Et (82,458) and Udon Thani (1,324,168). According to the HDC, the number of farmers who accessed healthcare services for injuries in each year varied; it was shown that in 2016, there was a total of 12,580,864, 663,729, and 41,350 farmers who visited healthcare services nationally, in Udon Thani province, and in Roi-Et province, respectively. The total number of registered farmers in Thailand recorded by the Ministry of Agriculture and Cooperatives in 2016 was 4,178,064. ^32^

**Table 3.** Health Data Centre (HDC) morbidity rates (per 100,000 farmers) of occupational diseases and injuries, and comparison of the number of farmers from the HDC database and the number of registered farmers in 2016.

Occupational disease	Area/region	Number of cases	Number of HDC farmers ^**†**^	Number of registered farmers ^**‡**^	Morbidity rate per 100,000 HDC farmers *
WMSDs	Nation	41,912	11,829,540	4,178,064	354.30
	Roi-Et	19	41,108	207,465	46.22
	Udon Thani	1,800	660,883	154,478	272.36
Noise- and heat-related diseases	Nation	25,370	24,293,821	4,178,064	104.43
	Roi-Et	71	82,458	207,465	86.10
	Udon Thani	1,326	1,324,168	154,478	100.14
Pesticide toxicity	Nation	4,293	12,157,336	4,178,064	35.31
	Roi-Et	6	40,993	207,465	14.64
	Udon Thani	184	663,389	154,478	27.74
Injuries	Nation	75,612	12,580,864	4,178,064	601.01
	Roi-Et	147	41,350	207,465	355.50
	Udon Thani	2,837	663,729	154,478	427.00
Lung diseases **	Nation	-	-	4,178,064	1,129.00
	Roi-Et	3,852	252,223	207,465	1,527.20
	Udon Thani	6,995	846,456	154,478	826.40

^†^ The number of planting farmers as per the occupational code from the Health Data Centre (HDC) database.

^‡^ The number of registered farmers of the Ministry of Agriculture and Cooperatives. ^32^

^*^ HDC morbidity rate per 100,000 HDC farmers.

^**^ There was no report available of occupational disease category by HDC of the nation.

**Figure 1.**
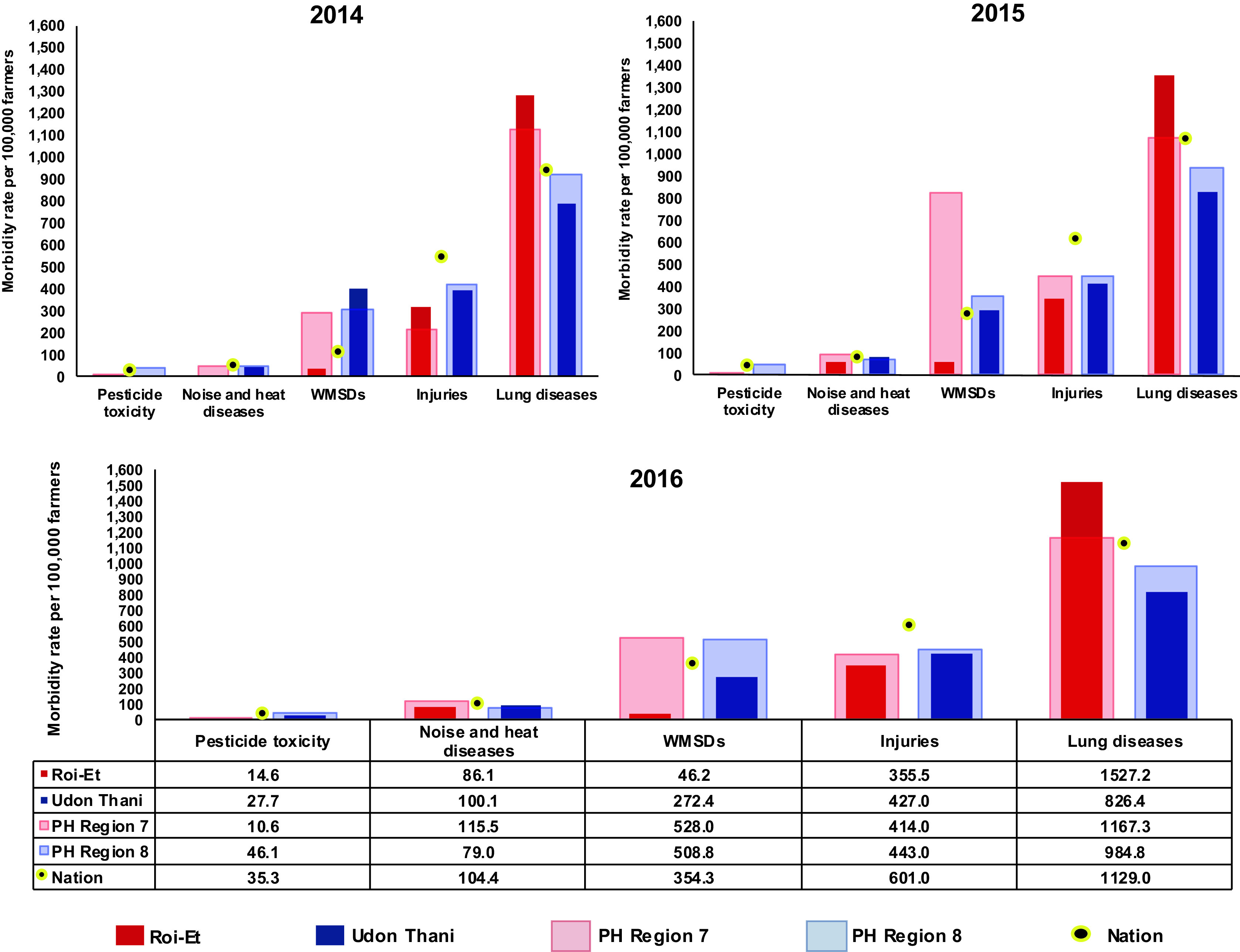
Morbidity rate per 100,000 farmers, classified according to occupational diseases and injuries recorded by the Health Data Center (HDC) of Thailand from 2014 to 2016.

The morbidity rate of occupational disease and injuries in Thailand, i.e., WMSDs, noise- and heat-related diseases, and pesticide toxicity and injuries (per 100,000 farmers), increased between 2014 and 2016, according to the HDC database. According to national results, the results of PH region 8 and Udon Thani supported this trend of increasing morbidity rate (as shown in Figure 1). Noise- and heat-related diseases and injuries have been increasing every year in all regions, which is also supported by the rates from Roi-Et province and PH region 7.

### Morbidity rate of work-related disease and injuries among farmers who visited healthcare service providers

This study analyzed the rate of chronic lower respiratory symptoms (J40-J47) as one category of lung diseases among farmers between 2014 and 2016. This was analysed in this manner following the categories of the top five prevalence of occupational diseases among crop planting farmers ^24^ as in Table 2. The disease was not directly reported as representative of a kind of occupational disease classified in the HDC database, but there was a reported data available as categories by age groups for 5-9 years, 10-14 years, 15-59 years, and 60 years or more, which could be accountable for the cases of workforce ages as agriculturists. ^22^ In 2016, the national rate of lung disease was 1,129.0 per 100,000 farmers, and the highest rate was found in Roi-Et (1,527.2), which was higher than that of the national total. The rate of lung disease was followed in order by the rates of injuries, WMSDs, noise- and heat-related diseases, and pesticide toxicity, respectively, and there was a higher morbidity rate in Udon Thani compared to Roi-Et. In Roi-Et, injuries were found to be at the highest rate (319.4 per 100,000 farmers) in 2014, which was followed by WMSDs (41.0), pesticide toxicity (11.4), and noise- and heat-related diseases (6.8), while the top-ranking rate of Udon Thani was that of WMSDs (405.3), which was followed by those of noise- and heat-related diseases (47.4), and pesticide toxicity (12.8), while the injuries rate was 400.0. In 2015, apart from injuries, lung disease (1,359.3 per 100,000 farmers) had the top-ranking morbidity rate of diseases among farmers in Roi-Et, followed by those of noise- and heat-related diseases (67.3), WMSDs (62.7), and pesticide toxicity (14.5), according to the HDC. Meanwhile, in Udon Thani, the morbidity rate of lung disease was the highest rate (831.6), followed by WMSDs (296.8), noise-and heat-related diseases (88.0). A similar trend was found during 2014-2016, in that the morbidity rates of Udon Thani, or PH region 8, in this year were mostly close to the national rate. In 2016, the morbidity rate of chronic lower respiratory symptoms (lung diseases) was the highest among all diseases and injuries in Roi-Et (1,527.2 per 100,000 farmers) and was also shown to be the highest among all diseases and injuries when compared to other regions and the nation ( Figure 1, see underlying data: TABLE A). ^49^

Even though the number of cases of diseases was stable, the number of people who had a recorded occupation of farmer in the HDC database and the number of registered farmers were different. Udon Thani as well as the nation always had a higher recorded number of farmers in comparison to the registered number of farmers, which contrasted with Roi-Et, as shown in Table 3.

In 2016, all rates of Udon Thani were higher than those of Roi-Et, i.e., with a 5.89, 1.16, 1.89, and 1.20-times higher rate of WMSDs, noise- and heat-related diseases, pesticide toxicity, and injuries, respectively.

Although the number of registered farmers of Roi-Et Province was higher than that of Udon Thani, the recorded cases or number of farmers to health service visits were lower compared to Udon Thani, for all diseases and injuries ( Table 3). The morbidity rate of disease was higher in Udon Thani in comparison to Roi-Et. It was found that the morbidity rates of the nation and Udon Thani Province had been increasing similarly.

When comparing the rate of occupational disease and injuries among registered farmers in 2016 to the rate of farmers who visited health service providers, the higher rate was found in the registered farmers for WMSDs, noise- and heat-related diseases, and injuries (pesticide toxicity was scarcely different), especially in Roi-Et, where the rate was 27.58 times higher for injuries and 16.32 times higher for WMSDs. Regarding those who identified as farmers when visiting Udon Thani health service providers, they had a 1.76 times lower rate of WMSDs and a 15.83 times lower rate of injuries than registered farmers. Similarly, the rates of noise- and heat-related diseases among those who identified as farmers in Udon Thani and Roi-Et provinces were about two times lower in the HDC database, in comparison to the rates of registered farmers.

### Morbidity rate of work-related disease and injuries of registered farmers visited health care providers in 2016

According to the secondary dataset of the health standard data structure (43 health files) and the number of agriculturists registered to the provincial agricultural office during 2014-2016, without duplicated registration of farmers or disease accounts, the analysis showed the number of registered farmers in 2014 to 2016 of Udon Thani Province was 154,478, while that of Roi-Et was 207,465 farmers (see underlying data: TABLE C). ^49^ By utilising the 43 health files data of the ICD-10 code of occupational cases and injuries during 2014 to 2016, it was found that there were 53,794 total cases (34.82%) of registered farmers who had accessed healthcare in Udon Thani, and 77,438 total cases *(*37.32% *)* in Roi-Et. Regarding the characteristics of those cases of registered farmers in Roi-Et and Udon Thani provinces, more than 60% were female. The largest proportion of cases in Roi-Et were working on rice plantations (98.26%), followed by cassava (1.12%), sugar cane (0.19%) and rubber (0.37%) plantations. Regarding cases in Udon Thani, most cases were working on rice plantations (89.56%), and the next highest proportions were working on the following plantations, i.e., cassava (4.97%), sugar cane (4.56), rubber (0.24%), and soybean/corn (0.67%). Almost 80% of them worked in relatively small farming areas of less than 4 acres. Most of them (about 70%) produced less than 10 tons (see underlying data; TABLE D). ^49^

The results of morbidity rates in 2016 are presented in Table 4. The ranking of morbidity rates of work-related disease among registered farmers was as follows: lung disease, WMSDs, noise- and heat-related diseases, and pesticide toxicity, i.e., 2781.7, 754.3, 221.7, and 8.7 in Roi-Et and 2,278.6, 479.1, 207.7, and 26.5 in Udon Thani, respectively. The rate of work-related injuries was more than four times higher compared to all rates of other diseases in Udon Thani and Roi-Et, as shown in Table 4.

**Table 4.** Morbidity rate (per 100,000 farmers) of occupational diseases and injuries among registered farmers of the provincial agriculture office in 2016 utilized from 43 health files data.

Diseases/injuries	Cases		Morbidity rate (95%CI)	
	Roi-Et	Udon Thani	Roi-Et	Udon Thani
WMSDs	1,565	740	754.3 (717.6-792.5)	479.1 (604.2-684.6)
Noise- and heat-related diseases	460	321	221.7 (201.9-242.9)	207.7 (203.3-306.6)
Pesticide toxicity	18	41	8.7 (5.1-13.7)	26.5 (16.8-46.3)
Injuries	20,341	14,025	9,804.6 (9,676.9-9,933.3)	9,078.9 (8,936.1-9,223.3)
Chronic lower respiratory tract disease (Lung diseases)	5,771	3,520	2,781.7 (2,711.3-2,853.3)	2,278.6 (2,204.8-2,354.3)

### The trend of work-related disease and injuries among registered agriculturists

From the 43 health files data, it was found that the morbidity rates among the registered agriculturists of Roi-Et were ranked in the following order, from highest to lowest, as follows: injuries, lung diseases, WMSDs, noise- and heat-related diseases, and pesticide toxicity, in every year from 2014 to 2016. The morbidity rate of registered agriculturists was not consistent with those who identified as farmers, as recorded by the HDC, in every year from 2014 to 2016. In 2016, registered agriculturists in Roi-Et had a higher morbidity rate of all diseases and injuries than Udon Thani, except pesticide toxicity. The rates and trends increased every year in this manner, except for pesticide toxicity, which had a lower rate in Roi-Et when compared to Udon Thani. In Udon Thani, the morbidity rates from the 43 health files were found to have the same ranking as Roi-Et for every health problem, and all those rates had increasing trends from 2014 to 2016.

**Figure 2.**
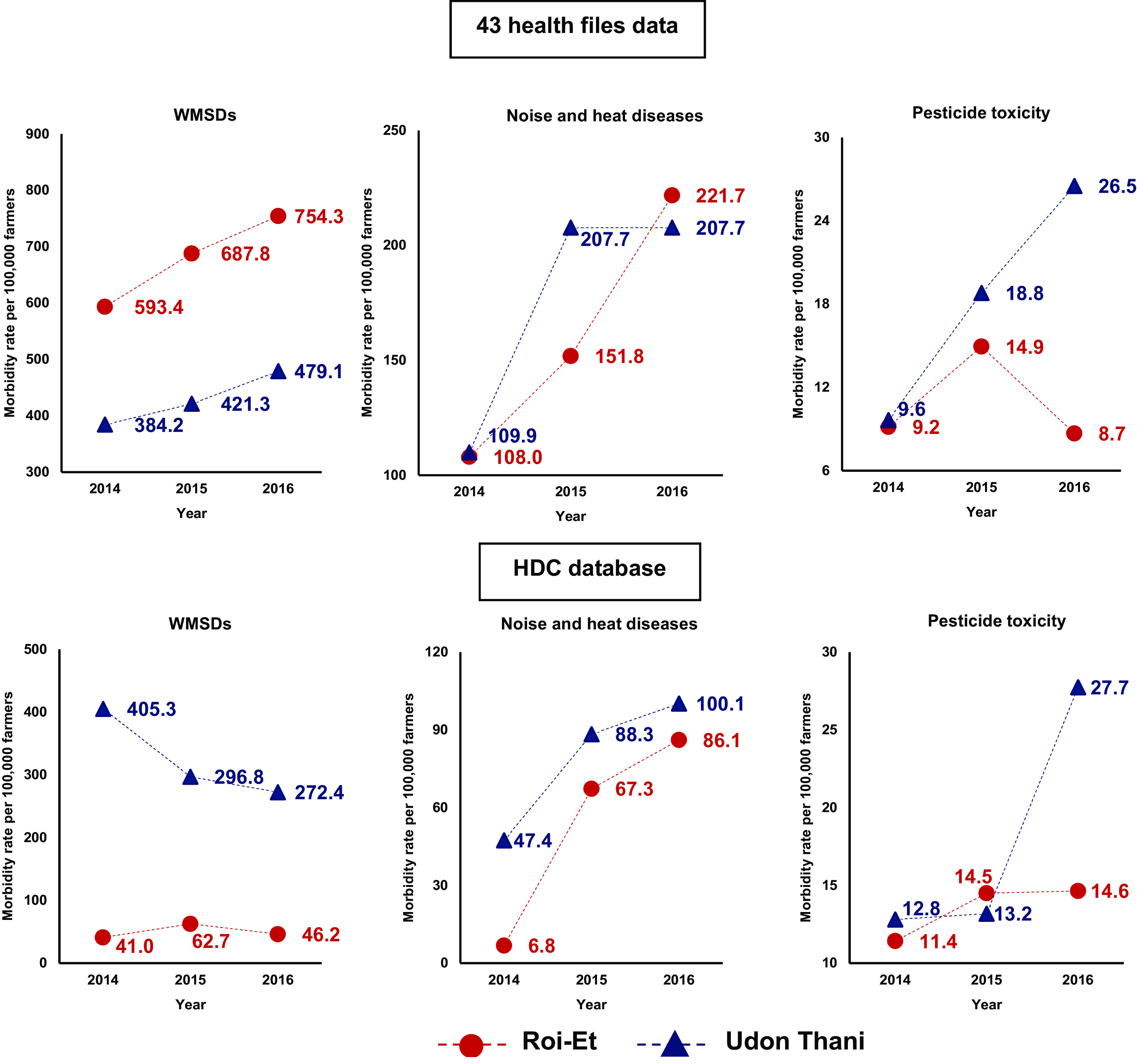
Trends of morbidity rate (per 100,000 farmers) from 2014-2016, classified by occupational disease, from the 43 health files data of registered agriculturist and the Health Data Centre (HDC) database of the HDC farmers.

The results from the HDC database showed that the morbidity rate of all diseases and injuries had an increasing trend, except WMSDs in Udon Thani, which remained constant as shown in Figure 2.

## Discussion

At present, the numbers of work-related diseases and injuries reported and cases among Thai farmers are likely to be lower than the true numbers because the health surveillance data of farmers or agricultural workers may not be accurate, as can be seen from the following findings and discussion.

By using registered farmers as the population for the morbidity rate calculation, which was different from using those who used a farmer occupational code as the population to calculate the rate from the HDC database, one could explain the inaccurate number of agriculturists found in healthcare service reports. The number of farmers from the HDC database may sometimes include duplicate recordings of farmers who visited healthcare providers more than once or sometimes be missing the occupational code identification of an agriculturist. That inaccurate number of farmers from the HDC database has led to incorrect results of morbidity rate. Therefore, for the next step of analysis, it was necessary to study registered agriculturists for accounted cases of occupational disease among agriculturists.

Those results of work-related diseases and injuries, excluding lung disease, among registered agriculturists, from the two case studies of Udon Thani and Roi-Et provinces from analysis of the HDC database and the 43 health files data, showed the same ranking of morbidity rate, which were injuries, WMSDs, noise- and heat-related diseases, and pesticide toxicity, respectively. Consequently, both Udon Thani and Roi-Et were possibly representative of national rates. For the case of lung diseases, the number of cases who had lung disease specific to the age group of between 15 to 59 years of age was analysis for the morbidity rate from HDC database, the disease was not further reported in the HDC database as occupational disease as mentioned before. It showed the highest morbidity rate from our analysis for chronic lower respiratory tract disease, or a specified lung disease among occupational diseases. However, when considering the morbidity rates (per 100,000 registered farmers) of diseases and injuries from 2014 to 2016 found in the 43 health files data, it was found that the highest morbidity rate from our analysis was not for a specified lung disease, which had not been found before with regard to occupational disease in the disease surveillance system of the Ministry of Public Health, Thailand. The high morbidity rate of occupational lung disease, which was not yet covered by the HDC database, might be explained by the fact that in disease diagnosis, it was not found to have an external cause, which would require recoding to Y96 to specify an occupational disease group.

Some other diseases related to exposure to agrochemical hazards were recommended from the previous studies, e.g., Parkinson’s disease ^38^; cancers of specific organs, e.g., lung, pancreas, lymphohematopoietic organs, bladder, prostate gland, ^39^ or thyroid ^40^; gestational diabetes mellitus (GDM) ^41^ or diabetes ^42^; etc. In covering those diseases to build up the surveillance system among agriculturists, some more investigation is still needed to confirm an association between each disease and a specific toxic substance used in pest control. Moreover, occupational lung disease should be considered in health surveillance of disease among farmers as per the previous report done by using a systematic review among Thai agriculturists. ^43^

Excluding lung disease, the three top-ranking rates across all regions and years were injuries and/or WMSDs, followed by hearing loss and diseases from heat, and pesticide toxicity, respectively. In addition, the rate of injuries was highest among all occupational diseases in Roi-Et, Udon Thani, and the nation. Udon Thani, which was chosen as a good representative province of PH Region 8, had a top-ranking rate which was the same as the nation, namely that of injuries, followed by WMSDs, noise- and heat-related diseases, and pesticide toxicity, respectively. These findings confirmed the ranking rate results found among registered crop farmers in Nong Bua Lamphu, one of the provinces in PH Region 8, Thailand; ^24^ however, all morbidity rates of WMSDs were more than 10 times lower than those found in other countries in Asia, ^2^ Canada, ^3^ and Europe. ^4^ With regard to noise- and heat-related disease, the rate found was 100 times lower compared to that of America, ^5^ ^,^ ^7^ and the rates of injuries and pesticide toxicity were found to be extremely low when compared to those found in Nepal ^10^ and China. ^16^ The closest morbidity rate to global rates was that of the cases of lung disease, which was more than two times lower than that of South Africa. This finding of low morbidity rates from the health database of Thai agriculturists when compared to global rates might be explained by underestimation of the morbidity rates of occupational disease and injuries.

In cases of toxicity from pesticides in 2016, the morbidity rate in Roi-Et Province was lower than previously reported among southern Roi-Et farmers during the same period, ^26^ which could be explained by local agricultural behaviors and the fact that pesticide toxicity report cases depended on the disease surveillance of each regional public health service. ^44^ Moreover, it was noteworthy that Roi-Et provincial-level case reports were very low in number compared to those of Udon Thani ( Table 3).

The accuracy of the health databases varies based on the hospital level; for instance, data from hospitals at the provincial level are more accurate than those at the district level and all collected data in this study are summations of sources of data at the provincial level, which are sent to the health data centre at the national level. On the other hand, inaccuracies and actual missing data exist in Thailand, which is a major problem of diagnosing occupational diseases with a coding of Y96, and is one main problem that has been reported before. ^44^ However, this study used a method of matching cases with an ICD10 diagnosis code of individuals identified by national ID to cases of those who had a registered occupation of agriculturist; both types of cases can be found in databases which present the situation of occupational disease among agriculturists in Thailand.

According to the analysis of ICD-10 utilized from the 43 health files standard among registered farmers, the morbidity rate of WMSDs in Roi-Et was around double that of Udon Thani. In contrast, the morbidity rate of WMSDs in Udon Thani, according to the HDC database, was around 10 times higher than that of Roi-Et. Regarding this comparison of morbidity rates of WMSDs, namely the HDC database versus the 43 health files data records, the trends were found to be unclear. This might be explained by the fact that case reports of WMSDs are not only specific to farming work, or they have multi-factor causes. ^43^ Another explanation might be the wrong or missing diagnostic coding of Y96, which signifies a specified occupational cause, as reported earlier regarding the health surveillance system in Roi-Et province. ^44^ However, from our data of the cases extracted from the 43 files of the most recent year, 2020 ^45^ it was confirmed that WMSDs had an increasing trend and a higher morbidity rate in Roi-Et than in Udon Thani province. Intriguingly, it was found that between the years 2014 and 2016, the province of Roi-Et had the fourth-highest tonnage of inland rice production in the country. ^46^ In comparison to Udon Thani, Roi-Et’s in-season rice yield was approximately 2-3 times higher. ^46^ This study discovered from the 43 health files data that the WMSDs rate in Roi-Et was greater than that in Udon Thani province, presumably because rice farming requires more body exertion in various agricultural activities throughout the year than farming of other crops, e.g., cassava and sugar cane, which is planted more in Udon Thani.

According to HDC database analysis, Udon Thani had a higher rate than Roi-Et with regard to injuries, and diseases caused by noise and heat and pesticide toxicity; increasing rates were also observed from 2014 to 2016 among Udon Thani and Roi-Et agriculturists. Those two occupational disease groups as well as injuries were confirmed as the major health problems among agriculturists in Thailand. ^43^

Infectious diseases, such as leptospirosis and melioidosis, had the highest prevalence in farmers, but there was no infectious disease group shown for occupational disease in the HDC database. This was not only the case in Thailand; a previous study in Poland showed a significantly higher rate in farmers who had been exposed to repeated tick bites, ^47^ which corresponded to annual reports in Greece from 2004–2015. ^15^ Moreover, brucellosis cases have been found in farmers and livestock breeders, with a high incidence rate of 7.1 per 100,000 population. ^15^

From the HDC database, it was found that the ranking of the morbidity rates, in Roi-Et, in 2014, excluding that of lung disease, were as follows: morbidity rate of injuries, followed by that of WMSDs, pesticide toxicity and noise- and heat-related diseases, respectively. However, during 2015 and 2016, the morbidity rates changed in order. Meanwhile, the ranking order of morbidity rates of Udon Thani (2014-2016) was the morbidity rate of injuries, followed by that of WMSDs, noise- and heat-related diseases and pesticide toxicity, respectively. That explains why Roi-Et was unable to provide good representative data because the province had not reported data as forecast, which contrasted with Udon Thani province in this study. The morbidity rates from the 43 files health data standard of Roi-Et province showed congruous results among the registered farmers. The highest rate was for work-related injuries, followed by WMSDs, hearing loss and heat-related disease, and pesticide toxicity, respectively. Similar results were found in Udon Thani, where rates of work-related injuries, WMSDs, and noise- and heat-related disease were similar and close to the national rates. Hence, these results reveal that the provincial data of Udon Thani can be representative of the nation. The consistency of the database is shown by the top three highest morbidity rates of disease from the previous study in crop farmers of Nongbualamphu province, which were shown to be WMSDs, noise- and heat-related disease, followed by skin irritation. ^24^ Moreover, the number of reported cases was very different between Udon Thani and Roi-Et, and the morbidity rate of pesticide toxicity in Roi-Et was dramatically lower than that of the previous report from 2016-2018 among registered farmers in Sakon Nakhon, a province in PH Region 8. ^25^ Those cases mentioning skin disease were suspect due to a combined effect in the group of pesticide toxicity, which is that of a reported acute symptom (irritant), and confirmed the previous studies. ^25^ ^,^ ^26^

Regarding agricultural productivity in the study area, the Office of Agricultural Economics reported that cassava, rubber, and rice had the highest productivity between 2016 and 2019, ^46^ as found in the case characteristics of this study. That could support results concerning the significant increase in morbidity rates of all occupational diseases and injuries in Roi-Et from 2014 to 2016; this increase was also found in Udon Thani, particularly in regard to pesticide toxicity. Pesticide toxicity has been shown to be a potential disease of the agricultural sector of Thailand, and as discussed, that problem was exacerbated by the trend of increasing pesticide imports, particularly herbicide imports, during the 5-year period from 2013 to 2017. ^48^ In comparison to Roi-Et province, Udon Thani has almost five times the production areas of cassava and Para rubber. ^46^ This study reveals that Udon Thani has a higher increasing trend of morbidity rate for pesticide toxicity than Roi-Et province. Hence, it is clear that herbicides are being used continuously to treat the rubber and cassava while it is being harvested.

These disputations with strong evidence suggest health surveillance of occupational disease among agriculture workers (informal workers) or Thai farmers by 1) realizing the diagnosis with a coding record of Y96, 2) improving the recording method of occupation to be used practically for agriculturists, and 3) promoting agriculture workers to register on the health database. It was more than interesting that some have mentioned other diseases should be added to ICD-10 for occupational disease, i.e., lung disease, skin disease and infectious disease, for the occupational surveillance system.

## Conclusions

The morbidity-rate ranking of work-related diseases and injuries, excluding lung diseases, among agriculturists in the upper northeast of Thailand was injuries, WMSDs, noise- and heat-related disease, and pesticide toxicity, respectively. Lung disease, which was not reported as an occupational disease of the HDC database, had the highest morbidity rate among occupational diseases in Roi-Et (2,781.7 per 100,000 farmers) and Udon Thani (2,278.6 per 100,000 farmers) from the 43 health files data. The national morbidity-rate ranking in 2016 (per 100,000 farmers) from the HDC database was injuries (601.0), work-related musculoskeletal disorders (WMSDs) (354.3), noise- and heat-related diseases (104.4), and pesticide toxicity (14.6), respectively. Those morbidity rates of Roi-Et and Udon Thani provinces were representative of the morbidity ranking of diseases of the nation. When comparing the morbidity rates of diseases of farmers from the 43 health files data to those of the HDC database, the closest rates were found in the pesticide toxicity of Roi-Et (26.5 per 100,000 farmers) and Udon Thani (27.7 per 100,000 farmers). The rates of WMSDs in Roi-Et (754.3 per 100,000 farmers) and Udon thani (479.1) from the 43 health files data were more than two times higher than those rates from the HDC database (Roi-Et: 46.2 and Udon Thani: 272.4 per 100,000 farmers), which was like those of noise-and heat-related diseases as well as that of injuries. All work-related diseases and injuries of the 43 health files data and almost all of those of the HDC data had increasing trends from 2014 to 2016. The number of farmers in the HDC database did not consistently reflect the number of registered farmers in the agricultural database. Situations of work-related diseases and injuries discovered among registered farmers reflect the health problems of Thai agriculturists, and the underestimation in the reported disease rate in the health database is explained by big data analysis, which showed that work-related cases with an identifying code of Y96 had rarely been reported among agriculturists. Therefore, recording an occupational cause when visiting health service providers should be promoted in the future, and Thai agriculturists should be supported in registration with healthcare databases for surveillance of various types of occupational diseases and injuries, including lung, skin and infectious diseases.

## Data availability

### Underlying data

The underlying data for this study is secondary data collected by the Health Data Centre and agricultural registries and is restricted due to ethical and data protection considerations. Under the ethical approval from the Human Research Ethics Committee of Khon Kaen University (No. HE592154), participants’ data was not allowed to be shared and the secondary data of health dataset and registered farmer dataset were utilized under restriction. The individual information or further reused inclusion data is not permitted under the ethically approved conditions for human research. This was confirmed under the data protection of human/participant privacy as no individuals gave explicit written consent that their identifiable data can be made publicly available. For more information, this research had no personal identified data for accessible because of the ethical issue that does not allow us to hold the personal data of each participant and cannot be shared any data after closing research.

The raw dataset collected under permission access and restriction use of ICD-10 codes are the 43 health files lists of Roi-Et and Udon Thani provinces in the period 2014 to 2016: file 10 – diagnosis (opd), file 15 – diagnosis (ipd), and file 19 – surveillance and the lists of registered agriculturists in Roi-Et and Udon Thani provinces during 2014-2016. To request access to the underlying data, please email the primary investigator (Sunisa Chaiklieng; csunis@kku.ac.th as the corresponding author. Applications will be reviewed on a case-by-case basis.

The intermediary data that can be de-identified without compromising anonymity are all presented already in the results and underlying data (TABLE A, B, C, D) and listed below.

Open Science Framework (OSF): Situations of work-related diseases and injuries among agriculturists in the upper northeast regions of Thailand. https://doi.org/10.17605/OSF.IO/2JPZM. ^49^

The project contains the following underlying data:

• TABLE A B C D ( Table 1 - data for Figure 1; Table B - data for Figure 2 that is related to HDC database; Table C - data for Figure 2 that is related to 43 Health files; TABLE D – data for characteristics of cases of work-related diseases and injuries)

Data are available under the terms of the Creative Commons Zero “No rights reserved” data waiver (CC0 1.0 Public domain dedication).
