# Supplementary material for: Situations of work-related diseases and injuries among agriculturists in the upper northeast regions of Thailand
Source: F1000Res. 2023 Jan 23;11:145. Originally published 2022 Feb 4. [Version 2] doi: 10.12688/f1000research.73221.2 (PMC10170183; doi:10.12688/f1000research.73221.2)
Supplement: Peer review history

# Peer review: Reviewer response for version 2

**DOI:** 10.5256/f1000research.142425.r161208

**Published:** 2023-02-02

**Authors:** Donald Cole

**Affiliations:** 1 Dalla Lana School of Public Health, University of Toronto, Toronto, ON, Canada

Lots of explanations and improvements to your paper - many thanks. Your paper could still benefit from some assistance with English language editing.

Is the work clearly and accurately presented and does it cite the current literature?

Partly

If applicable, is the statistical analysis and its interpretation appropriate?

Yes

Are all the source data underlying the results available to ensure full reproducibility?

Partly

Is the study design appropriate and is the work technically sound?

Yes

Are the conclusions drawn adequately supported by the results?

Partly

Are sufficient details of methods and analysis provided to allow replication by others?

Partly

Reviewer Expertise:

NA

I confirm that I have read this submission and believe that I have an appropriate level of expertise to confirm that it is of an acceptable scientific standard, however I have significant reservations, as outlined above.

# Response

**Published:** 2023-05-03

**Authors:** Sunisa Chaiklieng

**Affiliations:** Khon Kaen University Faculty of Public Health, Thailand

Dear Prof. Donald Cole

 This update version was improved from the second version for English proofreading/editing by University academic English specialist and native speaker. Thank you very much for your suggestion.

# Peer review: Reviewer response for version 2

**DOI:** 10.5256/f1000research.142425.r161209

**Published:** 2023-01-24

**Authors:** Pornchai Sithisarankul

**Affiliations:** 1 Department of Preventive and Social Medicine, Faculty of Medicine, Chulalongkorn University, Bangkok, Thailand

I have no more comments.

Is the work clearly and accurately presented and does it cite the current literature?

Yes

If applicable, is the statistical analysis and its interpretation appropriate?

Partly

Are all the source data underlying the results available to ensure full reproducibility?

Yes

Is the study design appropriate and is the work technically sound?

Partly

Are the conclusions drawn adequately supported by the results?

Yes

Are sufficient details of methods and analysis provided to allow replication by others?

Yes

Reviewer Expertise:

Occupational and environmental medicine/health, preventive medicine, public health

I confirm that I have read this submission and believe that I have an appropriate level of expertise to confirm that it is of an acceptable scientific standard.

# Peer review: Reviewer response for version 1

**DOI:** 10.5256/f1000research.76861.r142386

**Published:** 2022-07-08

**Authors:** Pornchai Sithisarankul

**Affiliations:** 1 Department of Preventive and Social Medicine, Faculty of Medicine, Chulalongkorn University, Bangkok, Thailand

1. Background - ..work-related diseases and injuries.. -- so the result should show or address injuries data
2. Results - should show key findings in numbers
3. Registered farmers may not be the true population at risk, as discussed in Conclusions; moreover, this bias may not be equal in the 2 provinces
4. Other potential biases are - discrepancy of diagnoses and codings of the 2 provinces and across the 3-year period, different quality of diagnoses and codings of different levels of hospitals, etc.  - these should be addressed and discussed
5. Be aware of the difference between 3-year report/statistics and 3 consecutive-year report/statistics
6. Neurotic hearing loss - I do not agree to use this term. There are sensorineural, conductive and mixed hearing losses.
7. Regarding each individual province, Roi-Et had an increasing trend for all diseases, for both data from the 43 health files data and data from the HDC, but a disease of Udon Thani had a clearly decreasing trend, -- I do not understand and am not sure which disease is " a disease of Udon Thani".

Is the work clearly and accurately presented and does it cite the current literature?

Yes

If applicable, is the statistical analysis and its interpretation appropriate?

Partly

Are all the source data underlying the results available to ensure full reproducibility?

Yes

Is the study design appropriate and is the work technically sound?

Partly

Are the conclusions drawn adequately supported by the results?

Yes

Are sufficient details of methods and analysis provided to allow replication by others?

Yes

Reviewer Expertise:

Occupational and environmental medicine/health, preventive medicine, public health

I confirm that I have read this submission and believe that I have an appropriate level of expertise to confirm that it is of an acceptable scientific standard, however I have significant reservations, as outlined above.

# Response

**Published:** 2022-12-20

**Authors:** Sunisa Chaiklieng

**Affiliations:** Khon Kaen University Faculty of Public Health, Thailand

**Dear Prof. Dr. Pornchai Sithisarankul**

 Thank you for your valuable comments, we have improved paper following your suggestions, as the revised version. The clarification for each question is following.  

 1. Background - work-related diseases and injuries - so the result should show or address

*Answer:* This results and discussion of paper was improved to address injuries data as suggestion.

*2. Results - should show key findings in numbers*

*Answer:* The results was improved to show key findings in numbers as suggestion.

 3. Registered farmers may not be the true population at risk, as discussed in conclusions; moreover, this bias may not be equal in the 2 provinces

*Answer*: The most recent agricultural census, conducted every ten years, was conducted in 2013. In each province of the nation, data collection will take place simultaneously by sending around 20,000 officers, 17,000 counting personnel/employees, 3,000 academic staff, and farmland holders/heads of each household to conduct interviews to compile fundamental data about agricultural operations. These details will be utilized to choose appropriate agricultural properties and conduct a meeting with qualified details (enumeration). By that reason, the registered farmers in Thailand are strongly representative agricultural farmers by an occupation, resulting on occupational hazards exposure and the true population at risk for this study, as we have done in the previous studies and method [24] [25] [28]. In addition, among the registered farmers, the real strong point is the linking of the identified cases to the full farmers population approved by the Provincial Agriculture Office agriculture database, that to show the magnitude of risks of the agricultural farmers.

 Moreover, the analysis in this study was accurate and have reliability by matching personality with the national ID to the health records of 43 files based on identifiers of individuals to another source of the registered farmers for Bigdata analysis. This method was done for previous studies [24, 25-26] which could show the risks of the Thai agricultural farmers on occupational diseases, particularly WMSDs, heat related illness or pesticide toxicity, that highly risks in agriculture were under-estimated from the previous report accounted 2-10 times of health database as in this study report. Any bias which might not be equal in the 2 provinces, we mentioned in the discussion to this serious and lack or difficulty to get support of recording occupational disease may have caused non-reporting as in the discussion.

 4. Other potential biases are - discrepancy of diagnoses and codings of the 2 provinces and across the 3-year period, different quality of diagnoses and codings of different levels of hospitals, etc. these should be addressed and discussed.

*Answer:* The discussion was added for those issues, it was discussed that the accuracy of the health databases varies on the hospital level; for instance, the provincial level is more accurate than the district level and all collecting data in this study are summation at the provincial level sources of data, where sending data to health data center of a country level. Whereas inaccuracies and actual missing data exist in Thailand is the major problem of diagnosing occupational diseases coding of Y96, which is one main problem as we discussed (Discussion section). However, this study method of analysis, we matched case of ICD10 diagnosis code to the registered agriculturist occupation (more by national ID. Both databases are the best representative for presentation the situation of Occupational diseases among agriculturists in Thailand.

 The only potential bias could be by quality of diagnoses and coding of different levels of hospitals which was addressed in discussion section according to the forecast which might occur and missing diagnostic coding in case of WMSDs, moreover, pesticide toxicity report cases depended on the disease surveillance of each regional public health service. ^44^

 5. Be aware of the difference between 3-year report/statistics and 3 consecutive-year report/statistics

*Answer*: We aware of the difference, all parts in this paper and underlying data were rechecked and replaced with the correct term to present in this paper, for example, from 2014 to 2016, in 2016, thank you for this recommendation.

 6. Neurotic hearing loss - I do not agree to use this term. There are sensorineural, conductive and mixed hearing losses.

*Answer:* This term was replaced with noise-induced hearing loss or shortly hearing loss following the HDC database and definition.

 7. Regarding each individual province, Roi-Et had an increasing trend for all diseases, for both data from the 43 health files data and data from the HDC, but a disease of Udon Thani had a clearly decreasing trend, I do not understand and no sure which disease is a disease of Udon Thani

*Answer:* This sentence was rewritten as; “All work-related diseases and injuries of the 43 health files data and almost all of those of the HDC data had increasing trends from 2014 to 2016.”

# Peer review: Reviewer response for version 1

**DOI:** 10.5256/f1000research.76861.r141342

**Published:** 2022-07-04

**Authors:** Donald Cole

**Affiliations:** 1 Dalla Lana School of Public Health, University of Toronto, Toronto, ON, Canada

For a global audience, the first paragraph is less relevant than the 2nd. I would suggest moving it down to a Thailand specific focus before the HDC paragraph.

 In the literature review paragraph, you might start by citing some of the reviews of the range of agriculture and health in general before going into each disease-disorder-injury type.

 Re the HDC, need some more background about its role in surveillance or health data in Thailand. 

 A case-control study does not usually result in prevalence estimates. Clarify how this might provide such an estimate.

 Good to have methods of identification of the above. Seems like preliminary work to this paper. Might it be better as an early part of methods?

 This work is clearly important for Thailand. How might it be important globally, in other MICs for example? How does it relate to the Global Burden of Disease work e.g. on low back pain? Something more generalizable as a scientific objective is needed. For example, it seems that there are two: one around work-related injury and disease surveillance methods, in which the paper makes a contribution; and one around differential burdens in different settings and exploration of reasons.

 Great use of a registered farmer - farmworker data set. Any sense what the breakdown would be i.e. proportions with different access rights to land? employers or employees?

 Not sure what you mean by "more real" agriculture? what is "less real" agriculture? How did you decide on the occupational codes in table 1?

 In some jurisdictions, farmer suicides from pesticides are considered work-related, as reflect the difficult situation of many farmers, with cash and debt squeezes which force them into suicide as one of few options.  And pesticides are associated with depression. Please justify your exclusion e.g. more of a narrow safety view of exposure.

 Can you provide some sense of the completeness of coding of health conditions etc. This is often the challenge with regards to occupational disease and occupational injury occurrence.

 Data analysis, last two sentences of first paragraph should be edited together. Formula "of" or "in" rather than "at" area of interest.

 I found it confusing to have the number of registered farmers in the same table as number of HDC farmers. the latter is the denominator for table 3 and Figure 1, while the former is the denominator for subsequent analysis shown in table 4, where this denominator of registered farmers should be included.

 Not sure that the interprovincial comparisons are as important globally as they may be internally for national and provincial policy makers. Less emphasis perhaps, unless going to speak to the heterogeneity across regions within countries globally, and some of the reasons for such heterogeneity e.g. industrial sector profiles, meteorological conditions, etc.

 Agree that under-reporting common in surveillance. More reference to this methodological literature, the strengths and weaknesses of different systems, particularly for work-related injuries and diseases is needed. 

 What factors might explain the increasing morbidity trends? Is more awareness, education and diagnosis occurring? more exposure?  Need some exploration of reasons. Any data sources that might inform this discussion?

 Currently much repetition of results here, without linkage to the global literature. How do the rankings here compare with other country rankings of morbidity among agriculturalists?  and why, do you think.

 Why do you think health service providers do not record occupation in Thailand? How does it compare globally? 

 Really appreciate the data availability statement and your work in this regard. The OSF tables are more other versions of the results, however.

Is the work clearly and accurately presented and does it cite the current literature?

Partly

If applicable, is the statistical analysis and its interpretation appropriate?

Yes

Are all the source data underlying the results available to ensure full reproducibility?

Partly

Is the study design appropriate and is the work technically sound?

Yes

Are the conclusions drawn adequately supported by the results?

Partly

Are sufficient details of methods and analysis provided to allow replication by others?

Partly

Reviewer Expertise:

Work and environment related conditions and interventions to deal with them. In particular, WMSD, pesticide and fungal disease surveillance.

I confirm that I have read this submission and believe that I have an appropriate level of expertise to confirm that it is of an acceptable scientific standard, however I have significant reservations, as outlined above.

# Response

**Published:** 2022-12-20

**Authors:** Sunisa Chaiklieng

**Affiliations:** Khon Kaen University Faculty of Public Health, Thailand

Dear Prof. Dr. Donald Cole

 According to your comments, we have much appreciate and have improved paper following your suggestions, as attached version. The clarification for each question as following. 

 1.  For a global audience, the first paragraph is less relevant than the 2nd. I would suggest moving it down to a Thailand specific focus before the HDC paragraph.

*Answer*: It was improved as your suggested, thank you [see Introduction]

 2.  In the literature review paragraph, you might start by citing some of the reviews of the range of agriculture and health in general before going into each disease-disorder-injury type.

*Answer*: The literature review paragraph was improved following your suggestion [see Introduction].

 3. Re the HDC, need some more background about its role in surveillance or health data in Thailand. 

*Answer*: Background of the role in the surveillance of health for the HDC in Thailand was added in Introduction (see Introduction)

 4.  A case-control study does not usually result in prevalence estimates. Clarify how this might provide such an estimate.

*Answer*: The uncleared sentence as question regarding the case-control study was improved as rewritten as “The case-control study of registered agriculturists in Khon Kaen confirmed that the noise-induced hearing loss cases from the 43 health files data had been diagnosed through the audiometer test of those agriculturists [28]," thank you for this comment for improvement.

 5. Good to have methods of identification of the above. Seems like preliminary work to this paper. Might it be better as an early part of methods?

*Answer*: The preliminary work or our previous investigation to estimate the morbidity rate of occupational diseases by using the big data of ICD-10 codes among registered agriculturists was the retrospective study as the references [24-26, 29]. The case-control study was designed in the on one study of the hearing loss to identify the risk factors among registered farmers [27]. The reference of method was cited in the method section for ICD-10 codes of occupational disease groups and for analysis of the morbidity rate, i.e, reference 24, the study among registered farmers of Nongboulamphu province, thank you for the suggestion.

 6. This work is clearly important for Thailand. How might it be important globally, in other MICs for example? How does it relate to the Global Burden of Disease work e.g. on low back pain? Something more generalizable as a scientific objective is needed. For example, it seems that there are two: one around work-related injury and disease surveillance methods, in which the paper makes a contribution; and one around differential burden in different settings and exploration of reasons.

*Answer*: From the big data analysis from the 43 health files is clearly identified cases of occupational disease to estimate the morbidity rate by extracted ICD-10 code of occupational disease, for example, code M545 of the registered farmers holding cultivating activity who was matching national ID to the accessed healthcare farmers. The previous study among crop farmers [reference 24] could show the number of low back pain cases of 4,814 cases in 2016 that reflected the highest morbidity rate of WMSDs (21.73%) among all occupational diseases in registered farmers. The study discovered also lung diseases, which was not reported in HDC database as the occupational disease.

 The real strong point of the paper is the linking the identified cases to the full farmers population and the outcome is showing the magnitude of risks of the agricultural farmers, which is a large part of the workforce in not only in Thailand but throughout globally. Even in highly developed countries hazards in agriculture are usually under-estimated, and negative outcomes not identified due to compensations mechanisms as we mentioned in the discussion continued from the background of Thai labor protection and compensation among agriculturists who are informal workers in Thailand. This study used two provinces of Roi-Et and Udon Thani which are two of the provinces among the top five provinces engaged in cultivating activities in the Northeast of Thailand and the major of planting area and numbers of farmers in Thailand. The population of agriculturists in those two provinces represented Thai agriculturist as the nature of the impact from agricultural work which was found to be similar across from other areas of Thailand because the main product of all regions in Thailand was rice. Therefore, this paper could make a contribution to the disease surveillance method of work-related injury and diseases as we improved written more focusing in the abstract and discussion and the conclusion.

 7. Great use of a registered farmer - farmworker data set. Any sense what the breakdown would be i.e. proportions with different access rights to land? employers or employees?

*Answer*: The most recent agricultural census, conducted every ten years, was conducted in 2013. In each province of the nation, data collection will take place simultaneously by sending around 20,000 officers, 17,000 counting personnel/employees,3,000 academic staff, and farmland holders/heads of each household to conduct interviews to compile fundamental data about agricultural operations. These details will be utilized to choose appropriate agricultural properties and conduct a meeting with qualified details (enumeration). That strongly confirm that the registered farmers in Thailand are representative agricultural farmers as occupation, resulting the most true population at risk on occupational hazards exposure for this study. Roi-Et and Udon Thani which are two of the provinces among the top five provinces engaged in cultivating activities in the Northeast of Thailand were selected as representative provinces. The major of planting area and numbers of farmers in Thailand located in Northeast of Thailand, and the main product of all regions in Thailand was rice as presented in Roi-et and Udon Thani.

 For accessing of healthcare, this study, by utilizing the 43 health files data of the ICD-10 code of occupational cases and injuries during 2014 to 2016, it was found that there were 53,794 total cases *(*34.82%) of registered farmers who had accessed healthcare in Udon Thani, and 77,438 total cases *(*37.32% *) *in Roi-Et, that was not much different of those provinces. For the cases visited of healthcare as shown as additional results, the characteristics of those cases of registered farmers in Roi-Et and Udon Thani provinces, the largest proportion of cases in Roi-Et were working on rice plantations (98.26%), as well as in Udon thani (89.1%). Those showed only in numbers of the difference because of cultivating activities were different during off-rice season as we discussed.  

 Thai’s agribusiness is clear content with contact farming laws which registered farmers are taken care of by government benefits such as price insurance. This study year have no statistics of agribusinesses number because agricultural census is providing the survey of agricultural holdings and will be report in 2023. The updated agricultural census was reported about statistics of agribusinesses which they provided by survey the registered farmer. At the present, there are no contact farming entrepreneur registered with the government in Roi-Et and Udon Thani.

 8. Not sure what you mean by "more real" agriculture? what is "less real" agriculture? How did you decide on the occupational codes in table 1?

*Answer*: Roi-Et and Udon Thani are two of the provinces among the top five provinces engaged in cultivating activities in the Northeast of Thailand (comprising more than 97.0% of agricultural holdings) as explained before, therefore, the word of “more real” was deleted to be clear from the design of this study.  

 For the occupational codes in table 1 was the recorded code of occupation in HDC database as we already explained in method section; All codes of farmers presented in Table 1 are the cultivating famers or involved activities in cultivated farming as the reference code of farmers from the Health Data Center (HDC) ( https://hdcservice.moph.go.th/hdc/main/index.php) and confirmed by the IT personal responsible of coding and the agriculturist expertise.

 9. In some jurisdictions, farmer suicides from pesticides are considered work-related, as reflect the difficult situation of many farmers, with cash and debt squeezes which force them into suicide as one of few options. And pesticides are associated with depression. Please justify your exclusion e.g. more of a narrow safety view of exposure.

*Answer*:  This study aware of the suicide from pesticide and we already excluded the cases not be accounted to the morbidity rate of pesticide toxicity as shown at notes in Table 2. In addition, the case of patient death during the study year which was accessed from file number 3 of the 43 health files were excluded from the visited cases in this study.

 10. Can you provide some sense of the completeness of coding of health conditions etc. This is often the challenge with regards to occupational disease and occupational injury occurrence.

*Answer*:  The occupational codes of diseases and injuries used in this study methods are referred to reference code used for Occupational diseases and injuries of HDC database that is the limitation of the secondary analysis. Therefore, those codes were covered as used for the occupational diseases and injuries from the 43 health files data. However, we can  provide codes found  from the previous studies of 43 health file data and published papers (in Thai) of ICD-10 codes of health conditions of agriculturists, for example; the lower respiratory tract disease (lung diseases): J62, J630-J638, J660-J679, J68, J40-J47, WMSDs: M00-M99, M542, M545, M548, M60, M624, M653, M792, M796, G560,  hearing loss and heat illness: H833, H903-H905, T670-T679, T68, and for pesticides toxicity (T600, T601, T602, T603, T604, T608, T609),  which recommended more to add specified skin diseases: L01, L02, L230-L239, L240-L249, L250-L259, L303, L502, L503, L55, L560- L569, L904. Those occupational code found and confirmed report from cultivated agriculturists [as Reference no 24, 25,26,29,47] as we discussed.

 11. Data analysis, last two sentences of first paragraph should be edited together. Formula "of" or "in" rather than "at" area of interest.

*Answer*: It was corrected as follow;

*Number of cases reported among registered agriculturists or farmers visited services in an area of interest* *Total number of farmers (registered agriculturists or farmers visited services) in an area of interest* 

 12. I found it confusing to have the number of registered farmers in the same table as number of HDC farmers. the latter is the denominator for table 3 and Figure 1, while the former is the denominator for subsequent analysis shown in table 4, where this denominator of registered farmers should be included.

*Answer*: The numbers of registered farmers of Roi-Et and Udon-Thani referred to Table 3 were corrected as presented for table 4.

 13. Not sure that the interprovincial comparisons are as important globally as they may be internally for national and provincial policy makers. Less emphasis perhaps, unless going to speak to the heterogeneity across regions within countries globally, and some of the reasons for such heterogeneity e.g., industrial sector profiles, meteorological conditions, etc.

*Answer*: Thank you for this point and that is agree with the design of this study and the results, we have improved the abstract and conclusion to be less emphasis of interprovincial comparison, but representative the rate or trend of morbidity rate of health problems of Thai agriculturist was presented, as shown in the Abstract and Conclusion. For the discussion in the morbidity rate of different, it is mostly pointed for WMSDs which showed the contradictory rate and ranking orders. The heterogeneity across regions within countries, the agricultural productivity while off-rice seasoning was more discussed also for pesticide toxicity.

  14. Agree that under-reporting common in surveillance. More reference to this methodological literature, the strengths and weaknesses of different systems, particularly for work-related injuries and diseases is needed. 

*Answer*:  We confirm the previous studies (24-25) and the preliminary results of the southern Roi-Et (26), of the under- reporting cases from the surveillance system of occupational diseases (44) as discussed. This study showed one pational disease in HDC database, by the method of extracted ICD-10 coded of J40-J47, could be done from the 43 health files data matching to the agriculturists.  The strength of Occupational diseases surveillance in the HDC database is the platform cases recorded from the Universal healthcare service which is usually covered for Thai population and farmers in Thailand. However, there were some weakness, 1) at time of this study, is that the recording of occupational disease was not mandatory but depended on the decision of hospital management and resources. Work-related disease effects on examination and treatment were not confirmed, for example, in the case of silicosis; it has symptoms similar to tuberculosis (TB), therefore, the report might be missing because it was not confirmed by silica dust exposure in the workplace from stone and sand, or mining or sculpture, or working in the cement industry. Moreover, for example lung diseases, infectious diseases, had not been included in the HDC database of Thailand as types of occupational disease. 2) The accuracy of the health databases varies based on the hospital level; for instance, data from hospitals at the provincial level are more accurate than those at the district level and all collected data in this study are summations of sources of data at the provincial level, which are sent to the health data centre at the national level.

 3) The occupational codes for farmers as shown in Table 1 in HDC database seems to be complicated for recording during routine work for healthcare providers. For this weakness, the second method to use the number of registered agriculturists can be solve the more strength method.

 The strength of the method utilizing the ICD-10 from the 43 health database are the following; 1) the morbidity rate of lung disease estimation which can be provide among registered farmers by matching personal ID of cases to those farmers in agricultural database.

 2) As we found the morbidity rate for WMSDs was the highest concerned diseased among all diseases, WMSDs ICD-10 codes in Table 2 were categorised in the health surveillance system, but they only allow for the knowledge of disease rates and cannot be used to identify the organs that are affected in WMSDs as we have shown from the recent study report [47; the WMSDs: codes of diagnosis were M00-M99, M542, M545, M548, M60, M624, M653, M792, M796, G560], 3) limitation of diagnosis which is depended on the accessed healthcare service level. That is equal from the HDC database and the 43 health files method. 3)  Another weakness to be concerned from the disease surveillance system of the use of ICD-10 code with occupational causes, for example, pesticide toxicity, a previous study in Roi-Et province found that ICD-10 identification and reports on the disease surveillance system with external code Y96 (work-related condition) were missing, as were reports in severe cases of pesticide poisoning with T-toxicity coding (44). Therefore, pesticide toxicity was shown to be underestimated in regard to occupation, particularly that of cultivating farmers, and the analysis utilised ICD-10 from the 43 health file data could include more cases without code Y-96 to estimate the rate.

 Thailand’s public health policy should include ICD-10 code L (skin disease) and code J (lung diseases) to identify the acute and chronic effects of pesticide toxicity among applicators, as recommended in our previous study of cultivating farmers in Sakon Nakhon province (26). By that considered ICD-10 code L, the stronger method of 43 health files data can provide manually extract code ICD-10 of the cases of the registered farmers.

 15. What factors might explain the increasing morbidity trends? Is more awareness, education and diagnosis occurring? more exposure?  Need some exploration of reasons. Any data sources that might inform this discussion?

*Answer*:  From both data source, and we provided more data as shown in underlying data, Table D, and the discussion continued to the conclusions, the trend of increasing rate of diseases was presented. It can be explained from the main factors of health hazards exposure from cultivation activity and types of plantations affecting the different diseases rates of the two province- case study as discussed for pesticides toxicity and WMSDs increasing trend in the Discussion as the follow.

 “Regarding agricultural productivity in the study area, the Office of Agricultural Economics reported that cassava, rubber, and rice had the highest productivity between 2016 and 2019 (48), as found in the case characteristics of this study. That could support results concerning the significant increase in morbidity rates of all occupational diseases and injuries in Roi-Et from 2014 to 2016; this increase was also found in Udon Thani, particularly in regard to pesticide toxicity. Pesticide toxicity has been shown to be a potential disease of the agricultural sector of Thailand, and as discussed, that problem was exacerbated by the trend of increasing pesticide imports, particularly herbicide imports, during the 5-year period from 2013 to 2017 (49)”.

 ….”However, from our data of the cases extracted from the 43 files of the most recent year, 2020 [47], it was confirmed that WMSDs had an increasing trend and a higher morbidity rate in Roi-Et than in Udon Thani province. Intriguingly, it was found that between the years 2014 and 2016, the province of Roi-Et had the fourth-highest tonnage of inland rice production in the country (48). In comparison to Udon Thani, Roi-Et's in-season rice yield was approximately 2-3 times higher (50). This study discovered from the 43 health files data that the WMSDs rate in Roi-Et was greater than that in Udon Thani province, presumably because rice farming requires more body exertion in various agricultural activities throughout the year than farming of other crops, e.g., cassava and sugar cane, which is planted more in Udon Thani.”

 16. Currently much repetition of results here, without linkage to the global literature. How do the rankings here compare with other country rankings of morbidity among agriculturalists? 

*Answer*: The discussion was improved by linking to the global literature and compared with the ranking of morbidity among agriculturist as in section of discussion as the follow; ”For noise and heat related disease, the finding rate was 100 times lower compared to of the America (5, 7), which is similarly lower direction found in the rates of injuries and pesticide toxicity when compared to those found in Napal (10) and China (16). The closest rate to globally was that for the cases of lung disease which is lower than 2 times lower than in the south Africa. This finding too low morbidity rate from the health database of Thai agriculturists when comparing to those global rates might be explain the underestimation of the morbidity of the occupational disease and injuries. As we have a real strong point of the paper from the method from 43 health file big data analysis linking the identified cases to the full farmers population and the outcome is very showing the magnitude of risks of the agricultural farmers, which is a large part of the workforce not only in Thailand but throughout Asia and globally.

 17. Why do you think health service providers do not record occupation in Thailand? How does it compare globally?

*Answer*:  By HDC, the occupation code is available for case visited for healthcare service as we use the code of farmers involved plantation activities during the period of in this study, however, the many codes available to be selected to be represented farmers occupation as shown in Table 1 which is complicated for the healthcare providers to add in the record. There are also codes of unemployed, general employed, underling person, or no occupation, that could be selected for this filed, which made missing/misclassification in database. The previous study of pesticide poisoning reports among showed incomplete records of occupational cases in **45.14% of all cases**, and misclassification case report of agriculturists as occupation code was found in **37.03% of cases in Skon Nakhorn** from our previous case-control study *[Chaiklieng, S.; Khamjantarat, P.; Suggaravetsiri, P.* *Factors as*sociated *with disease from pesticides toxicity among planting farmers in Sakon Nakhon Province. In:* *Proceedings of The 9 ^th^ National Conference in Toxicology (NCT9)*, *Update in toxicology for safe life*. *25-26 September 2019, 109-21].* Moreover, there were key concerns regarding public health personnel being responsible for multiple tasks, without any understanding of occupational disease codes, and never having received training for occupational disease recording [44].

 As you agreed of under-reporting, and I am sure that even in highly developed countries, hazards and risks in agriculture are usually under-estimated, but negative outcomes not identified due to many reasons, i.e., lack of legal, control and compensations mechanisms. By those reasons, almost no recently update report to compared since 2004 after one study mentioning to that occupational history which was frequently neglect occupational history-taking by the physicians, and the physician had completed an occupational history-taking from only **27.8%** **of patients** (Politi et al. Occupational Medical History Taking. JOEM 2004;46:550-5), which is very low and also lower rate compared to our previous finding in Thailand [44].

 18. Really appreciate the data availability statement and your work in this regard. The OSF tables are more other versions of the results, however.

*Answer*:  We added more available results in Table D (underlying data): Characteristics of cases of work-related diseases and injuries among registered agriculturists in Roi-Et and Udon Thani provinces, in the upper northeast of Thailand. All data presented in Table A-D could be useful to learn more in numbers and rates or trend of occupational disease and injuries from this case study. The characteristic of cases of registered farmers, the numbers of cases from 43 health files data and the HDC database and the farmers or registered farmers in each year from our big data analysis during the study period could be useful to understand by other analysis or presentation of the readers or the researchers, because we did not show all rates presentation of the previous years (2014- 2015) in the paper, excepted 2016.  Importantly, the data described in this study are accessible from the corresponding author upon request. Due to confidentiality concerns, the data are not publicly available, as mentioned in the paper.
